# Wild rice-associated *Vibrio* promotes plant growth and exhibits genomic and phenotypic plasticity for plant adaptations

**DOI:** 10.1128/msystems.00910-25

**Published:** 2025-10-27

**Authors:** Kirti Kulanthaivel, Natarajan Rameshkumar

**Affiliations:** 1 Biosciences and Bioengineering Division, CSIR-National Institute for Interdisciplinary Science and Technology (NIIST) 91565, Thiruvananthapuram, Kerala, India; 2 Academy of Scientific and Innovative Research (AcSIR) 550336, Ghaziabad, India; University of South Florida Morsani College of Medicine, Tampa, Florida, USA

**Keywords:** plant-associated *Vibrio*, PGPR, plant-associated lifestyle, rhizosphere adaptation, diazotroph

## Abstract

*Vibrio* is an important marine heterotroph, primarily studied for its pathogenesis or symbiotic relationship with marine organisms and humans. However, little is known about the association of vibrios with plants in brackish environments and their potential benefits. To address this knowledge gap, we focused on *Vibrio porteresiae* MSSRF30^T^ and brackish-grown Pokkali rice as our research subjects for this study. MSSRF30^T^ displays multifaceted plant beneficial traits, including nitrogen fixation, 1-aminocyclopropane-1-carboxylate (ACC) deaminase production, and zinc and tricalcium phosphate solubilization. Further, MSSRF30^T^ efficiently colonizes the host roots and significantly improves the Pokkali rice growth in nitrogen-replete and nitrogen-limiting brackish conditions, highlighting its plant growth-promoting ability, a trait previously not well recognized in vibrios. Additionally, MSSRF30^T^ can utilize various carbon-rich substrates derived from plant roots, demonstrating its metabolic adaptation to the plant rhizosphere niche. Using *in planta* root transcriptome analysis and whole-genome sequencing, we provide the first insights into how MSSRF30^T^ interacts with Pokkali rice in brackish conditions. Additionally, we have identified several genome features for a plant-associated lifestyle, previously unreported in this genus. These features include plant expansin, PEP-CTERM surface anchoring with exopolysaccharides, plant-associated Hrp-type three secretion system, ACC deaminase production, PQQ-independent glucose dehydrogenase pathway for phosphate solubilization, plant-derived sugar/organic acids utilization operons, carbohydrate utilization loci, and specific plant depolymerizing CAZymes. Notably, MSSRF30^T^ lacks key genome features critical for the animal association. Overall, this study adds new knowledge in the field of *Vibrio* biology, especially *Vibrio*-plant beneficial interactions, a relationship largely underexplored.

**IMPORTANCE:**

The genus *Vibrio* comprises over 150 species of marine heterotrophic bacteria, many of which are opportunistic pathogens affecting humans and marine animals. Most research has predominantly focused on pathogenic *Vibrio* species, often overlooking the significance of other *Vibrio* species inhabiting other ecological niches, such as plants, a relationship largely uncharacterized. This study focused on *V. porteresiae* MSSRF30^T^ and its relationship with brackish-grown Pokkali rice. We discovered that MSSRF30^T^ possesses multiple plant growth-promoting traits, effectively colonizes roots, and enhances plant growth in brackish conditions. Additionally, MSSRF30^T^ possesses several genome features commonly associated with plant-microbe interactions, previously unrecognized in *Vibrio* species, and lacks features typically associated with animal interactions, underscoring its specialized adaptation for plant niches. For the first time, this study highlights the beneficial interactions between *Vibrio* and plants, emphasizing their role in promoting plant growth and health in brackish environments.

## INTRODUCTION

Bacteria engaged in mutualistic or commensal relationships with plants often benefit the host by supplying key plant nutrients, controlling phytopathogens, and increasing fitness to abiotic stress (1). Furthermore, these positive interactions can sometimes improve plant health and productivity (2). Hence, there is a tremendous interest in studying the plant-associated bacteria of various plants for their potential plant-beneficial functions and possible applications in crop improvement and productivity (3). In this regard, much of our key understanding of beneficial plant-bacterial interactions comes from studies primarily focused on plants and their rhizobacterial partners originating from terrestrial environments. In contrast, there is limited knowledge about the beneficial plant-bacterial interactions in brackish environments, a transition zone between marine and terrestrial environments, that hold agricultural significance.

With research interests in coastal agriculture, a 16S rRNA metagenomic study was conducted, specifically looking at habitat-specific plant-beneficial rhizobacterial communities of Kagga, a native salt-tolerant rice variety traditionally cultivated in brackish environments of Kumta, Karnataka, Southern India. Surprisingly, our analysis revealed a higher abundance of *Vibrio*-specific operational taxonomic units (OTUs) in the metagenomic data sets of Kagga rice (4). Notably, these *Vibrio*-specific OTUs were significantly enriched more in the rice root compartments (1.98%) than in the rhizosphere (0.9%) (4), signifying a close yet uncharacterized relationship between the *Vibrio* species and its plant hosts. Similarly, several culture-independent and culture-dependent bacterial community studies also reported the frequent occurrence and abundance of diverse *Vibrio* species in the rhizosphere and roots of various salt marsh plants, mangroves, and seagrasses grown in different brackish environments (5–16). Moreover, some of the *Vibrio* isolates were taxonomically classified as novel *Vibrio* species, namely *Vibrio rhizosphaerae* (17), *V. porteresiae* (18), *V. mangrovi* (19), and *V. plantisponsor* (20), were isolated from the rhizosphere or roots of mangrove-associated wild rice (*Porteresia coarctata*), *V. palustris* from the leaf of *Arthrocnemum macrostachyum*, and *V. spartinae* from the root of *Spartina maritima* (21). These findings indicate that the association between *Vibrio* species and plants is widespread in brackish environments. Nevertheless, little is known about the beneficial interactions of *Vibrio* species on plants in brackish environments.

The genus *Vibrio* represents a diverse group of marine heterotrophs, encompassing over 150 recognized species (https://lpsn.dsmz.de/genus/vibrio), many of which are opportunistic pathogens that affect humans and aquatic marine animals, while a few are identified as symbionts (22). As a result, most studies have primarily focused on *Vibrio* species that either cause severe diseases in humans, such as cholera caused by *V. cholerae* (23, 24) and vibriosis caused by *V. parahaemolyticus* (25) and *V. vulnificus* (26), or in aquatic marine animals, including coral bleaching caused by *V. coralliilyticus* (27) and shrimp vibriosis caused by *V. harveyi*, *V. anguillarum*, *V. alginolyticus*, *V. penaeicida*, and *V. parahaemolyticus* (28, 29), or engage in symbiotic relationships, such as *V. fischeri* with bobtail squid (30). This has led to a substantial body of literature on animal/human-associated *Vibrio* species covering different aspects, including their ecology, genomes, genetics, cell-cell communications, physiology, and mechanisms of pathogenesis or symbiosis. Nevertheless, these studies often overlook the importance of other *Vibrio* species inhabiting new ecological niches, such as plants, whose roles are not yet fully understood. Furthermore, specific genetic and phenotypic adaptations that enable *Vibrio* species to interact and thrive in a plant habitat successfully remain largely unknown.

To address this critical knowledge gap, we used *Vibrio porteresiae* MSSRF30^T^ (18) as a representative plant-associated rhizobacterium for this study. Through the integration of phenotypic analyses, whole-genome sequencing, and *in planta* transcriptomic profiling, we probed the plant-associated lifestyle and growth-promoting potential of MSSRF30^T^ in salt-tolerant Pokkali rice. Our results provide new insights into the genomic and functional adaptations of MSSRF30^T^ that possibly govern its plant-mutualistic association, representing the first report to characterize plant-beneficial traits in *Vibrio*.

## RESULTS AND DISCUSSION

### MSSRF30^T^ promotes plant growth under brackish conditions

The ability of MSSRF30^T^ to mediate plant growth was assessed in salt-tolerant Pokkali rice under a brackish environmental condition, mimicking the physiological state of brackish Pokkali rice fields.

**Fig 1:**
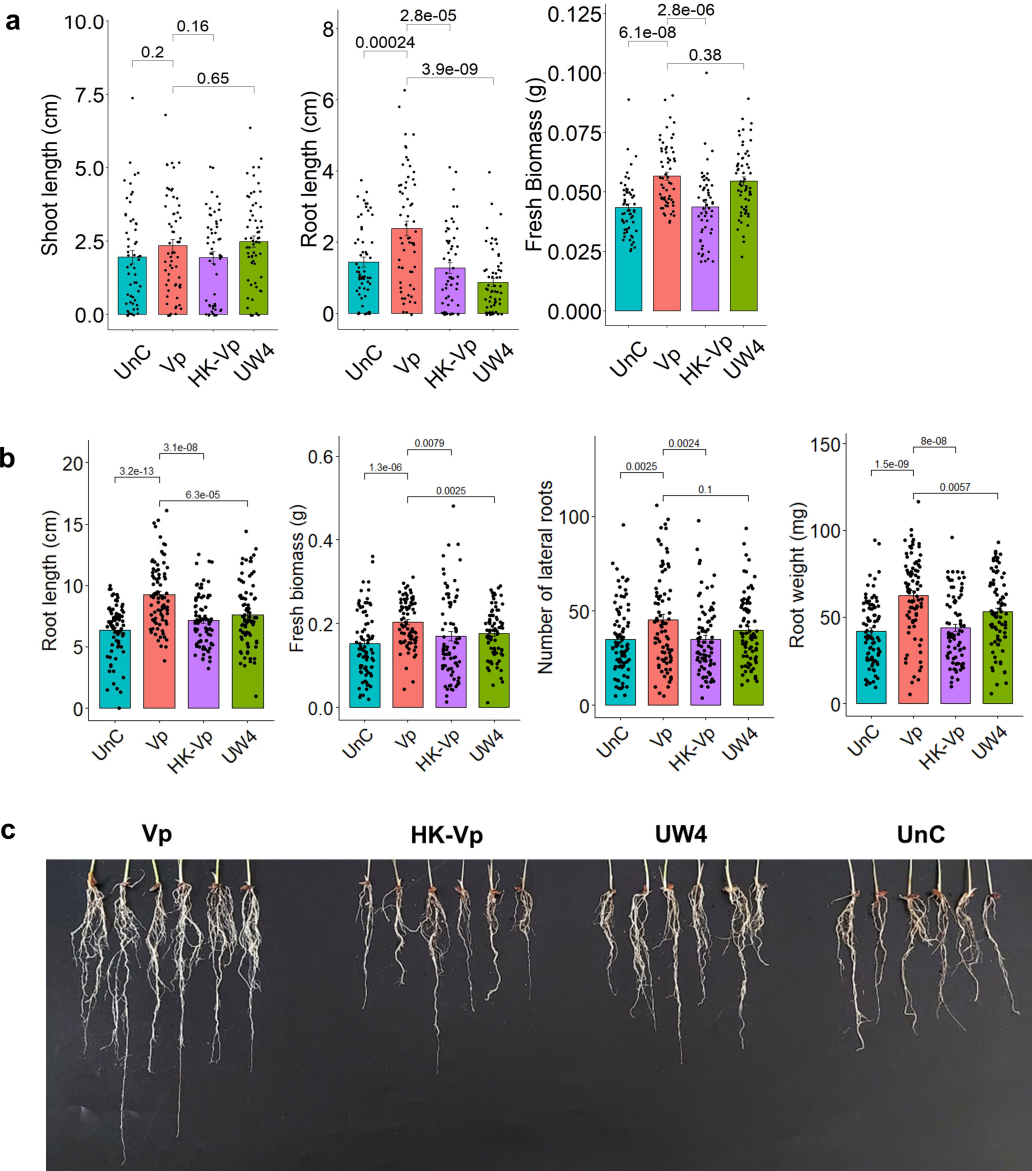
MSSRF30^T^ promotes the Pokkali rice growth. (**a**) The quantified plant growth parameters (shoot and root lengths, and biomass) of 10-day-old seedlings under each condition. Measurements were taken from approximately 20 plants per biological replicate. The average sample size was 59 seedlings. (**b**) Quantified plant growth parameters, including root length, fresh biomass, the number of lateral roots, and root weight, from a plant growth experiment conducted over 20 days using pots. The average sample size consisted of 83 seedlings. (**c**) Representative images of roots from six plants per condition, illustrating the effect of MSSRF30^T^ on the Pokkali rice root health. Seedlings were subjected to one of four treatments: incubation with MSSRF30^T^ (Vp), heat-killed cells of MSSRF30^T^ (HK-Vp), *P. putida* strain UW4 (positive PGPR), or left uninoculated (UnC). Error bars represent the standard error of the mean across three independent experiments in panels **a** and **b**. Statistical significance was assessed using an independent Student's *t*-test, with *P*-values < 0.001 indicating significant differences.

To begin, three-day-old sterile germinated Pokkali seeds were treated with MSSRF30^T^ cell suspensions (≥10^8^ cells) for one hour. After treatment, the seeds were aseptically transferred to petri plates containing sterile filter papers (refer to the method section for more details). To maintain a brackish environmental condition, filter papers with MSSRF30^T^-treated and untreated control seeds were regularly moistened using sterile plant nutrient solutions containing 20% natural seawater (PN-N^+^-20%NSW). After 10 days of incubation, the total biomass (*P*-value = 6.1emsystems00910-2508) and root length (*P*-value = 0.00024) were significantly increased in MSSRF30^T^-treated seeds compared to those treated with *Pseudomonas putida* strain UW4, heat-killed cell suspensions of MSSRF30^T^, and untreated control seeds (Fig. 1a). Similar results were observed in repeated experiments (data not shown). Following this observation, a plant growth experiment was conducted in pots using sterile vermiculite-quartz sand mixtures (2:1) as growth substrates. The inoculation procedure was carried out as previously described, and rice plants were grown in PN-N^+^-20%NSW. After 20 days of incubation, MSSRF30^T^-treated plants demonstrated a significant increase in the total biomass, root weight, root length, and the number of lateral roots compared to seedlings treated with UW4, heat-killed cells of MSSRF30^T^, or no bacterial treatment (Fig. 1b and c). These findings suggest that MSSRF30^T^ has the ability to enhance the Pokkali rice growth in brackish environmental conditions.

**Fig 2:**
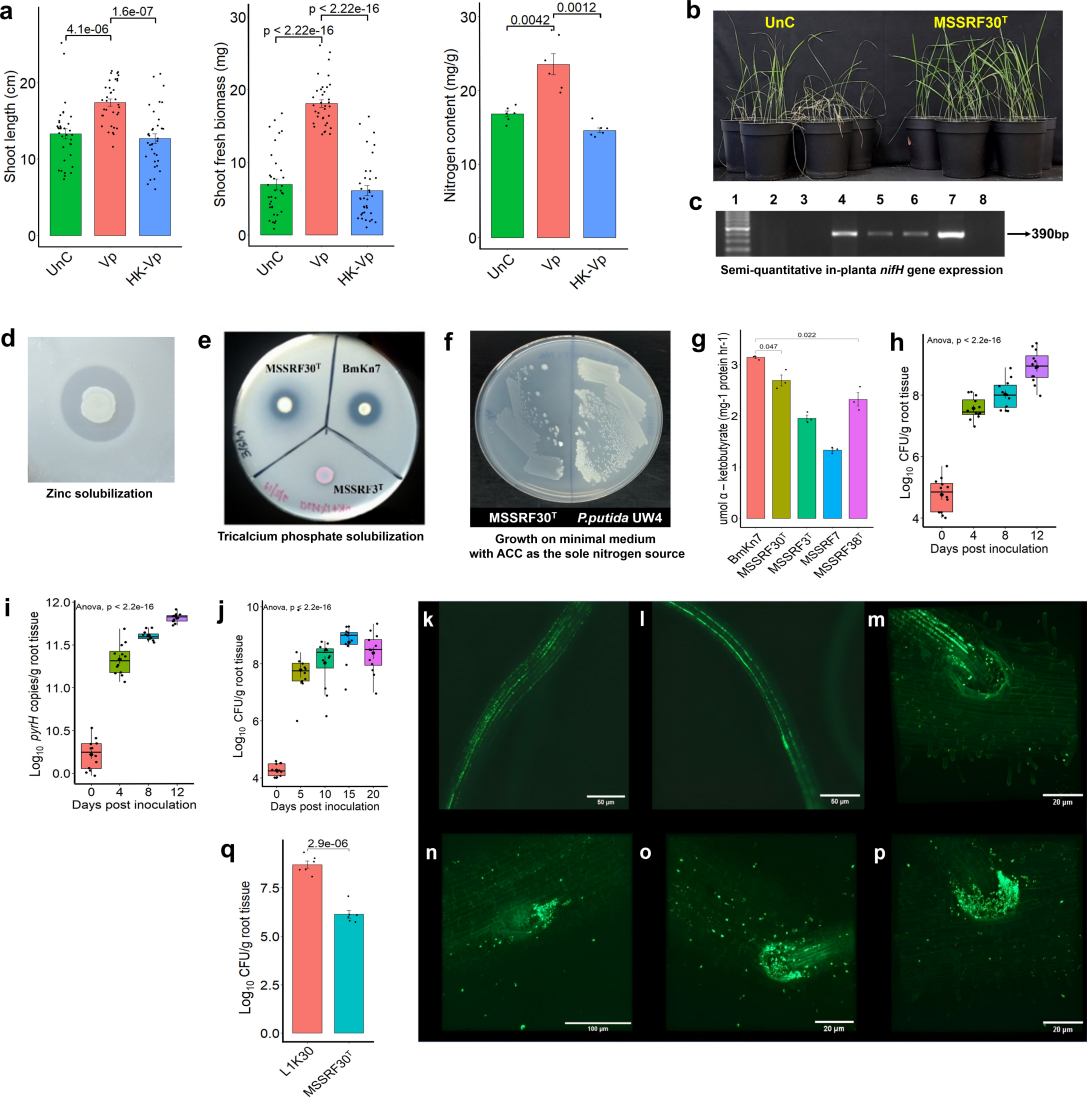
MSSRF30^T^ promotes plant growth under nitrogen-limiting brackish conditions, exhibits PGPR traits; ACC deaminase activity, and inorganic zinc and phosphate solubilization, and demonstrates robust root colonization. (**a**) The quantified plant growth parameters of shoot length, fresh biomass, and nitrogen content of plant samples treated with MSSRF30^T^ (Vp), heat-killed cells of MSSRF30^T^ (HK-Vp), and left uninoculated (UnC) under nitrogen-limiting (N^−^) brackish conditions. Error bars represent the standard error. Nitrogen content values are the average of six samples (each sample comprising shoots pooled from six plants for improved accuracy in Kjeldahl estimation). Shoot length and biomass values are based on 35 individual plants from two independent experiments. (**b**) Uninoculated and MSSRF30^T^-inoculated plants under nitrogen-limiting (N^−^) brackish conditions. (**c**) The 390-bp amplicon of the *nifH* gene from MSSRF30^T^ was detected using semi-quantitative RT-PCR, confirming nitrogenase expression and possible potential for nitrogen fixation. The legend for the labels is as follows: 1,100 bp ladder; 2 and 3, from roots grown in plant nutrient solution supplemented with NH_4_Cl; 4, from nitrogen-free Jensens broth; 5 and 6, from roots grown in plant nutrient solution devoid of nitrogen; 7, DNA of MSSRF30^T^ (positive control); 8, negative control. (**d**) Zinc solubilization by MSSRF30^T^. (**e**) Phosphate solubilization on Pikovskaya’s agar by MSSRF30^T^, with *Burkholderia* strain BmKn7 (positive control) and *Vibrio rhizosphaerae* MSSRF3^T^ (negative control). (**f**) Growth on modified M9 minimal media using ACC as the sole nitrogen source, and (**g**) ACC deaminase activity of *Burkholderia* strain BmKn7, MSSRF30^T^, *V*. *rhizosphaerae* MSSRF3^T^, *V*. *rhizosphaerae* MSSRF7, and *V*. *mangrovi* MSSRF38^T^, measured 48 hours post-inoculation. Quantification of MSSRF30^T^ (in CFU/g) recovered from colonized roots (**g**) and the corresponding log_10_ *pyrH* gene copies per gram of root tissue (from qPCR) (**h**), over 12 days. (**j**) CFU/g recovery from MSSRF30^T^-colonized roots was monitored over 20 days, indicating persistent root association. Confocal laser scanning microscopy images showing the root colonization pattern of MSSRF30^T^; within intercellular spaces along the root maturation zone (**k** and **l**), intercellular spaces of lateral roots (**m**), lateral root emergence sites (**n**), and lateral root junctions (**o** and **p**). These images highlight preferential colonization by MSSRF30^T^ at key root development sites. (**q**) Comparative CFU/g recovery of *Azoarcus* sp. L1K30 (positive control) and MSSRF30^T^ from the surface-sterilized roots of 5 days post-inoculation. One-way ANOVA was conducted on day-wise data to assess significant differences across time points (*P* < 2.2 × 10^−16^). Statistical significance was determined using an independent Student’s *t*-test (*P* < 0.001). Error bars represent the standard error of the mean across three (**g**) and two (**h–j** and **q**) independent experiments.

MSSRF30^T^ can fix atmospheric nitrogen (18), and additional experiments were performed to determine whether MSSRF30^T^ supports the Pokkali rice growth under a nitrogen-limiting brackish environmental condition. For this purpose, we used a similar experimental setup as previously described above, except the rice plants were grown in PN-N^−^-20%NSW (devoid of nitrogen, N^-^). This result showed a significant enhancement in the shoot biomass, shoot length, and shoot nitrogen content in plants treated with MSSRF30^T^ compared to the untreated control plants (Fig. 2a and b), suggesting that MSSRF30^T^ could promote the growth of Pokkali rice in nitrogen-limiting brackish environmental conditions. Furthermore, enhanced plant growth is likely achieved by providing fixed nitrogen to the host through the fixation of atmospheric nitrogen, as reported in several well-characterized plant-associated diazotrophs (31, 32). To support this view, a semi-quantitative *in planta nifH* gene expression assay was performed under both nitrogen-limitation and nitrogen-replete brackish environmental conditions. The result showed that MSSRF30^T^*nifH* transcripts were positively detected in rice root samples grown under nitrogen-limiting brackish conditions but absent in samples grown under nitrogen-replete brackish conditions (Fig. 2c). This suggests that MSSRF30^T^ can actively express *nifH* in Pokkali rice roots when nitrogen is limited and regulates its expression based on nitrogen availability in their growth environment.

Furthermore, MSSRF30^T^ is capable of solubilizing insoluble inorganic zinc and tricalcium phosphate (TCP), converting them to soluble forms by acidifying the growth medium through the production of gluconic acid (Fig. 2d and e; Fig. S1a and b). Additionally, MSSRF30^T^ produces 1-aminocyclopropane-1-carboxylate (ACC) deaminase (Fig. 2f and g; Fig. S1c and d), an important enzyme that modulates plant stress ethylene and promotes plant growth under abiotic stress (33–36). Notably, these findings reveal that MSSRF30^T^ possesses unique plant-beneficial traits not previously well documented in *Vibrio* species. Furthermore, the enhanced growth promotion observed in MSSRF30^T^-treated plants under a brackish environmental condition is likely attributed to its multifaceted plant growth-beneficial traits. Similar findings have been well documented in several terrestrial plant-associated PGPR strains (35–38).

Next, we investigated the root colonization ability of MSSRF30^T^, an important indicator of potential plant interactions, which is rarely studied in *Vibrio* species. Pokkali rice seedlings were grown up to seven days in clerigel, and then aseptically transferred to sterile phytajars containing PN-N^+^-20%NSW for this analysis. To this setup, MSSRF30^T^ cells were added, and at various time points, the roots were collected, and two methods were used to quantify MSSRF30^T^: (i) qPCR targeting MSSRF30^T^-specific *pyrH* gene and (ii) recovering of MSSRF30^T^ cells following the standard serial dilution plating method. Axenically grown plants were harvested every four days, and the colonization of MSSRF30^T^ is increased up to 12 days, reaching up to 10 log_10_ colony forming units (CFU) per gram weight of fresh roots (Fig. 2h). Quantification using qPCR showed consistent gene increase over 12 days, with significant differences between sampling points, reaching approximately 12 log_10_ copies of *pyrH* on the 12th day (Fig. 2i). The results from both methods indicated that the number of MSSRF30^T^ cells significantly increased from day 0 to day 12 post-inoculation, including an extended experiment conducted over 20 days (Fig. 2j). Furthermore, the colonization of Pokkali roots was examined microscopically using GFP-tagged MSSRF30^T^. Confocal microscopy revealed that GFP-tagged MSSRF30^T^ colonized the roots, preferentially on the root surfaces, root hairs, and intercellular spaces along the root maturation zone (Fig. 2k and l). Additionally, colonization was observed in the intercellular spaces of lateral roots (Fig. 2m), at the sites of lateral root emergence (Fig. 2n), and at the junctions of lateral roots (Fig. 2o and p; Movie S1). This colonization pattern closely resembles that of well-studied plant-associated PGPR strains (39, 40). The endophytic nature was also revealed by determining the CFUs of MSSRF30^T^ from the Pokkali rice roots after intensive surface sterilization (Fig. 2q). Overall, these results suggest that MSSRF30^T^ efficiently interacts with and multiplies in the roots of Pokkali rice under brackish conditions.

Several studies have reported on the association of vibrios with marine plants, including those found in salt marshes and mangroves (5–15). However, none of these studies have investigated the ability of vibrios to promote plant growth or provided evidence of their possessing multiple plant growth-promoting traits (PGPR). To the best of our knowledge, we demonstrate through our studies for the first time that plant-associated vibrios may play a crucial biological role in supporting plant health and growth in brackish environments.

### Genome features

We sequenced and annotated the complete genome of MSSRF30^T^ to understand its genomic basis of beneficial plant growth traits. The genome is 5,498,609 base pairs in size, has a GC content of 44.8%, consists of two chromosomes, and does not contain plasmids (Table S1a).

We analyzed the subsystem features of MSSRF30^T^ available in the RAST server in comparison to animal-associated vibrios to assess how the genomic composition of MSSRF30^T^ may reflect specific adaptations to a plant-associated lifestyle. This analysis revealed that a significant number of the protein-coding genes was classified under the functional category of metabolism. This included genes related to amino acid transport and metabolism, carbohydrate transport and metabolism, and inorganic transport and metabolism (Table S1b). These findings indicate that MSSRF30^T^ has specialized adaptations for metabolizing various plant-derived substrates, a trait often observed in the plant-associated rhizobacterial genomes (41).

### Genome information related to plant-associated lifestyle

#### Plant growth functions

##### Nitrogen fixation

**Fig 3:**
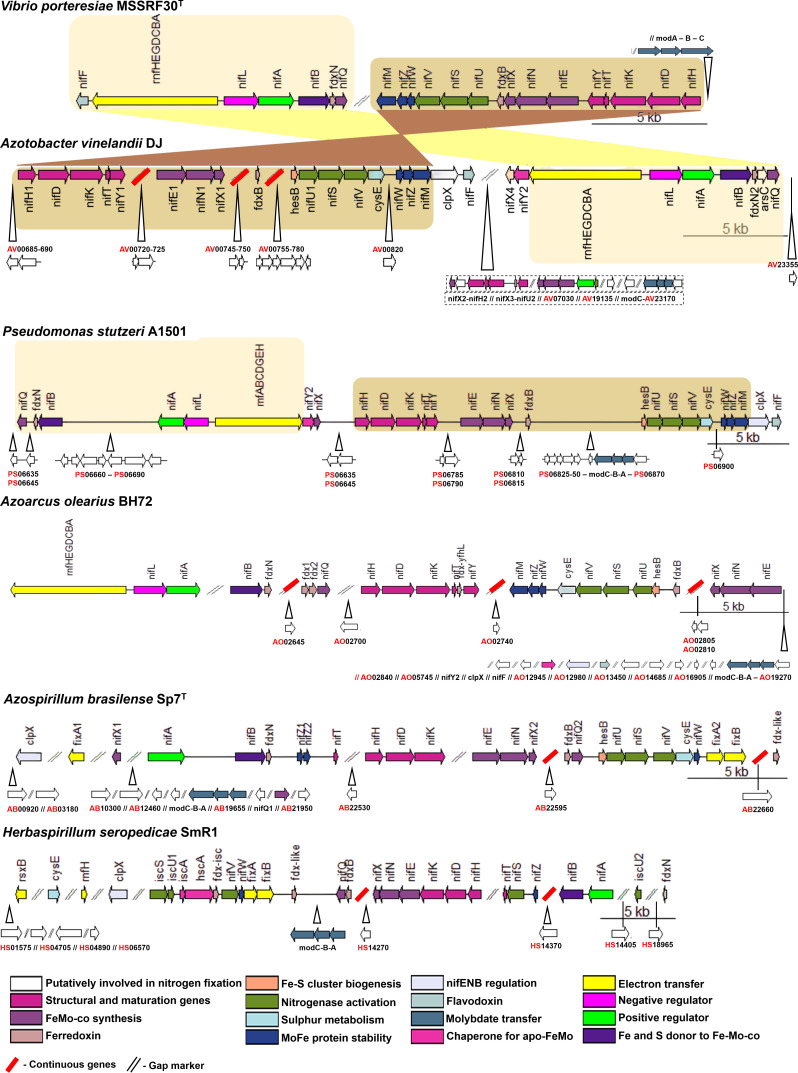
The *nif* gene cluster of MSSRF30^T^ and its synteny with other well-characterized *nif* gene clusters of plant-associated model diazotrophs.

MSSRF30^T^ is able to promote plant growth and fixes atmospheric nitrogen under a nitrogen-limiting brackish environmental condition (Fig. 2a and b) (18). Accordingly, MSSRF30^T^ carries a complete genetic system for nitrogen fixation (*nif*), including 19 *nif* genes encoded in a 31.87 kb region and distributed across three gene clusters located on the second chromosome (Fig. 3). Cluster one contained genes majorly involved in electron transport to nitrogenase (*nifF*, *rnfABCDGEH*, and *fdxN*) and positive/negative transcriptional regulations of *nif* regulon (*nifAL*). Cluster two carries the main genes encoding the molybdenum-iron dependent nitrogenase complex (*nifHDK*), iron-molybdenum cofactor (FeMo-co) biosynthesis (*nifYENXUSV*), and nitrogenase maturation (*nifMZ*). Cluster three encodes genes primarily involved in molybdenum transport to nitrogenase (*modABC*). Other important genes that participate in nitrogen fixation are shown in Table S1c.

The general *nif* organization of MSSRF30^T^ (clusters one and two) closely resembles the *nif* genetic system of *Azotobacter vinelandii* DJ (two clusters) and *Pseudomonas stutzeri* (one contiguous cluster) A1501, two well-characterized diazotrophs (42, 43). Notably, additional protein-coding genes predicated between the core nif genes of both DJ and A1501 were found absent in MSSRF30^T^, suggesting that MSSRF30^T^ probably involves a limited set of *nif*-associated genes for nitrogen fixation, considering a higher energy cost involved in this process (44). In support of the latter view, our transcriptome analysis of MSSRF30^T^ under an in vitro nitrogen-free condition revealed that all *nif* genes associated with clusters one and two transcribed significantly (Table 1), suggesting their potential involvement in nitrogen fixation.

**TABLE 1:** The list of significantly upregulated genes associated with nitrogen fixation during the growth of MSSRF30^T^ in nitrogen-free Jensen's broth

Protein ID	Annotation	Gene	Log2 fold change	*P*-adjusted value
*nif* gene cluster 1				
WP_261896644.1	Flavodoxin	*nifF*	8.63	1.57E−69
WP_261896646.1	RnfH family protein	*rnfH*	5.39	0.002069
WP_261896647.1	Electron transport complex subunit E	*rnfE*	5.75	5.16E−06
WP_261896648.1	RnfABCDGE type electron transport complex subunit G	*rnfG*	5.15	1.63E−09
WP_261896649.1	RnfABCDGE type electron transport complex subunit D	*rnfD*	7.96	7.73E−07
WP_261896650.1	Electron transport complex subunit RsxC	*rnfC*	7.95	9.61E−07
WP_261896651.1	RnfABCDGE type electron transport complex subunit B	*rnfB*	9.10	1.40e−09
WP_261896652.1	Electron transport complex subunit RsxA	*rnfA*	7.52	2.49E−08
WP_261896653.1	Nitrogen fixation negative regulator NifL	*nifL*	4.66	3.59E−06
WP_261896654.1	*nif*-specific transcriptional activator NifA	*nifA*	4.30	5.63E−08
WP_261896655.1	Nitrogenase cofactor biosynthesis protein NifB	*nifB*	8.05	2.23E−07
WP_261896656.1	4Fe-4S dicluster domain-containing protein	*fdxN*	9.30	5.88E−10
WP_261896657.1	Nitrogen fixation protein NifQ	*nifQ*	5.74	1.95E−07
*nif* gene cluster 2				
WP_261896700.1	Peptidylprolyl isomerase	*nifM*	6.44	1.43E−05
WP_261896701.1	Nitrogen fixation protein NifZ	*nifZ*	7.53	2.31E−12
WP_261896702.1	Nitrogenase-stabilizing/protective protein NifW	*nifW*	7.03	1.18E−13
WP_261896703.1	Homocitrate synthase	*nifV*	7.52	2.31E−12
WP_261896704.1	Cysteine desulfurase NifS	*nifS*	9.40	3.67E−14
WP_261896705.1	Fe-S cluster assembly protein NifU	*nifU*	9.85	1.03E−15
WP_261896706.1	Ferredoxin III, *nif*-specific	*fdxB*	27.43	7.03E−10
WP_261896707.1	NifB/NifX family molybdenum-iron cluster-binding protein	*nifX*	7.91	1.74E−09
WP_261896708.1	Nitrogenase iron-molybdenum cofactor biosynthesis protein NifN	*nifN*	8.40	3.79E−08
WP_261896709.1	Nitrogenase iron-molybdenum cofactor biosynthesis protein NifE	*nifE*	8.76	6.25E−09
WP_261896710.1	Hypothetical protein	–^*a*^	5.14	2.94E−04
WP_261896711.1	NifB/NifX family molybdenum-iron cluster-binding protein	*nifY*	7.02	1.12E−04
WP_261896712.1	Putative nitrogen fixation protein NifT	*nifT*	5.16	3.20E−05
WP_261896713.1	Nitrogenase molybdenum-iron protein subunit beta	*nifK*	7.11	9.66E−11
WP_261896714.1	Nitrogenase molybdenum-iron protein alpha chain	*nifD*	9.08	1.40E−09
WP_261896715.1	Nitrogenase iron protein	*nifH*	10.37	2.49E−13

^*a*^ "–" indicates unknown.

##### ACC deaminase

MSSRF30^T^ is positive for ACC utilization and ACC deaminase production (Fig. 2f and g). Accordingly, the MSSRF30^T^ genome carries putative genes encoding the synthesis (*acdS* gene, WP_261892913.1) and positive transcriptional regulation (*acdR*, belonging to the leucine-responsive regulatory protein family, WP_261892914.1) of ACC deaminase, a previously unreported genetic feature in the genus *Vibrio*. As observed in other *acdS*-positive Gram-negative plant-associated rhizobacterial genomes (45, 46), the regulatory *acdR* gene was located adjacent to *acdS* in MSSRF30^T^. Additionally, no mobile genetic elements, such as transposases or integrases, were identified in close proximity or flanking regions of the *acdS-acdR* gene locus, suggesting that no recent horizontal gene transfer event of *acdS* has occurred in MSSRF30^T^. Furthermore, the positive acquisition of *acdS* in MSSRF30^T^ might be because of its long-term adaptation toward the plant growth environment, where the expression of *acdS* could play a significant functional role in mutualistic plant interaction under abiotic stress (33–36). In support of this view, *acdS* was absent in most of the animal, coral, and algal-associated *Vibrio* genomes (data not shown), and an increase in *acdS* gene expression (log_2_ FC = 3.26) was observed in MSSRF30^T^ during its early Pokkali rice root colonization under nitrogen-limiting brackish conditions (Fig. S2; Table S2). Additionally, MSSRF30^T^ promotes Pokkali rice growth under a tested nitrogen-limiting brackish condition (Fig. 2a and b). These results indicate that acdS in MSSRF30^T^ could be functional and play an essential role in plant host interactions in nitrogen-poor brackish growth environments.

##### Inorganic phosphate solubilization

Gluconate and 2-ketogluconate are the two most important organic acids associated with inorganic TCP solubilization in agricultural soils (47–50). MSSRF30^T^ exhibits a strong ability to solubilize the insoluble TCP to its soluble form by secreting gluconic acid, thereby enhancing the phosphorus availability in the extracellular medium (Fig. 2e; Fig. S1a and b), a potential PGPR trait previously not reported in the genus *Vibrio*. Consistent with this finding, the MSSRF30^T^ genome harbors potential enzymes (Table S1e), including the flavin adenine dinucleotide (FAD)-dependent glucose dehydrogenase belonging to the glucose-methanol-choline (GMC) oxidoreductase superfamily (three copies) and a gluconolactonase, which participates in the oxidation of glucose to gluconolactone and then to gluconate. Furthermore, the presence of C-type cytochrome, a redox-active heme-containing protein (three copies) adjacent to each GMC oxidoreductase, suggests its possible role in the proper functioning of the GMC oxidoreductases (51). Two copies of membrane-bound gluconate dehydrogenase that catalyze the conversion of gluconate to 2-ketogluconate were also identified. More importantly, neither pyrroloquinoline quinone (PQQ)-dependent periplasmic glucose dehydrogenase nor the PQQ-cofactor encoding genes that facilitate the direct oxidation of glucose to gluconate, identified in several well-characterized efficient TCP-solubilizing rhizobacteria, including *Pseudomonas* and *Burkholderia* (49, 50), were encoded in MSSRF30^T^. Strikingly, FAD-dependent glucose dehydrogenase/GMC oxidoreductase was lacking in several *Vibrio* genomes (data not shown). These genomic findings suggest that MSSRF30^T^ might rely on the PQQ-independent glucose dehydrogenase pathway (i.e., FAD-dependent glucose dehydrogenase) to produce gluconate from glucose and solubilize the insoluble TCP. A similar genetic mechanism of phosphate solubilization was reported in *Collimonas pratensis* strain PMB3 (51).

### Secretion system

The genome of MSSRF30^T^ harbors two dedicated protein translocation secretion systems, Type III (T3SS) and VI (T6SS) secretion systems, and their respective effector proteins. This finding possibly suggests the potential capability of MSSRF30^T^ in mediating specific plant host interactions and interfering with eukaryotic and prokaryotic cellular functions, as seen in (52, 53).

#### T3SS

**Fig 4:**
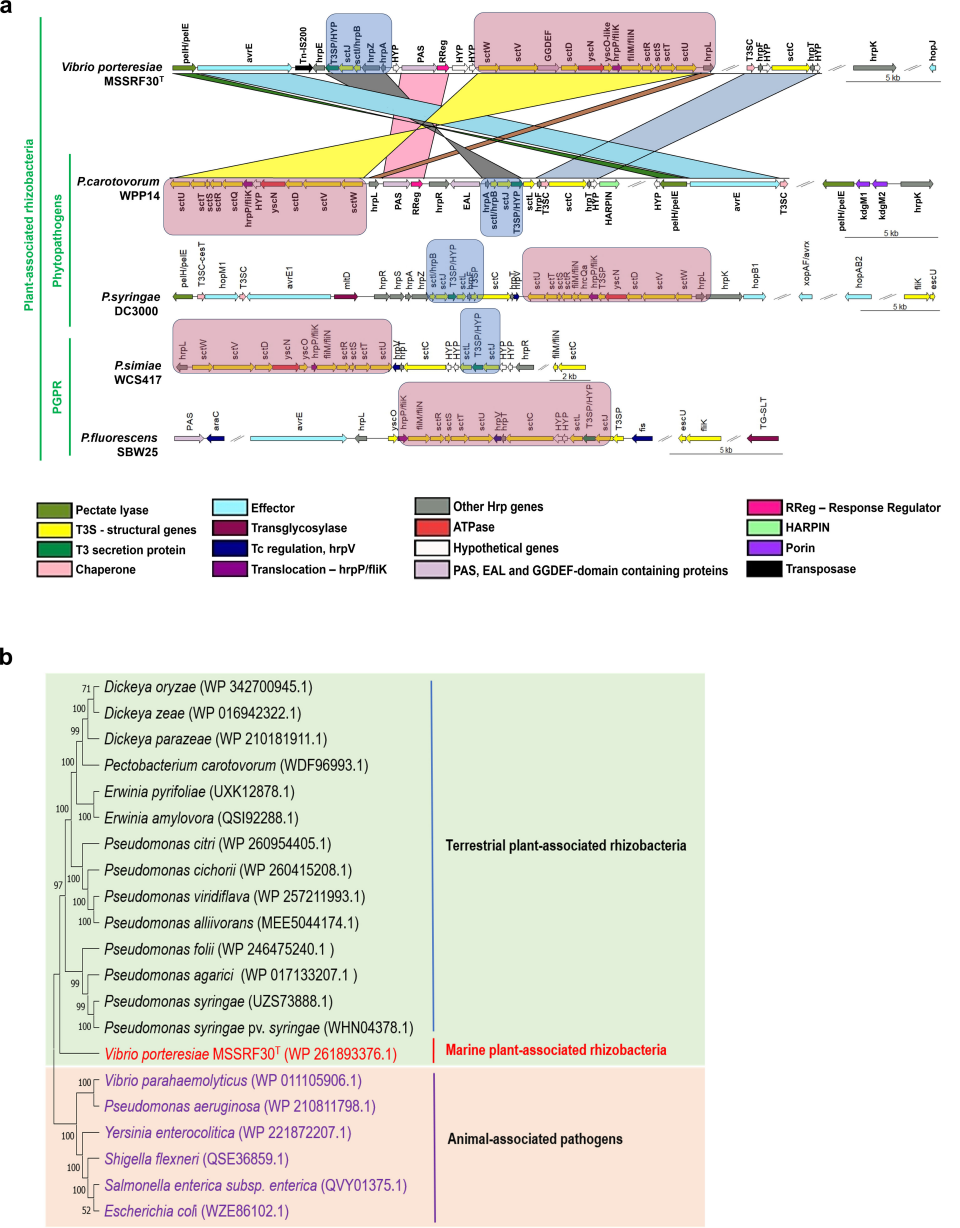
(**a**) Plant-associated Hrp-type T3SS gene cluster of MSSRF30^T^ and its synteny with other T3SS encoding plant-associated rhizobacteria, including plant pathogens and plant beneficial bacteria. T3SS of MSSRF30^T^ and *P. carotovorum* shared show close synteny, and the gene clusters containing *sctJ, sctI, hrpZ, hrpA, sctW, sctV, sctD, yscN, hrpP/fliK, sctR, sctS, sctT, sctU, and hrpL* are inverted. The order of *hrpF, hrpT, sctC,* and effector gene *avrE* is conserved. *P. syringae* and *P. simiae* retain a majority of these genes with a similar linear arrangement. *P. fluorescens* has the most divergent order of genes; however, many essential T3SS genes are retained, including *avrE*. *P. simiae* lacks the *avrE* effector, which seems to follow a non-homogeneous distribution pattern. (**b**) Phylogenetic analysis based on *sctC*. MSSRF30^T^ clusters with terrestrial plant-associated bacteria, including PGPR and plant pathogens, but are more distantly related to animal-associated bacterial pathogens. The significance of each branch is indicated by the bootstrap value (as a percentage) calculated for 1,000 subsets.

MSSRF30^T^ possesses a repertoire of 31 genes organized in a single large cluster located on chromosome two, along with two orphan genes on chromosome one encoding for T3SS (Fig. 4a; Table S1f). Importantly, this T3SS gene cluster shared a higher sequence homology and gene arrangement with the hypersensitive response and pathogenicity (*hrp*) apparatus of known plant pathogens (Fig. 4a) rather than the injectisomes of animal/human pathogens. Furthermore, phylogenetic analysis of the well-conserved T3SS outer-membrane secretin component (SctC) showed that MSSRF30^T^ T3SS forms a unique branch, separate from the T3SS cluster comprising several plant-associated rhizobacteria, including known phytopathogens and well-characterized PGPR, and is distantly related to the T3SS cluster consisting of animal and human pathogens (Fig. 4b). These results suggest that MSSRF30^T^ T3SS represents a yet-uncharacterized new phytopathogenic Hrp-type T3SS family member and a genome feature previously not reported in marine vibrios. Notably, a repetitive extragenic palindromic (REP)-associated tyrosine transposase (RAYT, WP_261893332.1) was predicted in the T3SS gene cluster interspaced between *avrE* and *hrpE*, indicating a possible acquisition of T3SS locus horizontally by MSSRF30^T^. Studies have shown that T3SS is vital in maintaining a pathogenic or symbiotic relationship between bacteria and eukaryotic hosts (52). MSSRF30^T^ possesses multiple PGPR traits and promotes Pokkali rice growth under a nitrogen-limiting brackish condition, suggesting a possible role of T3SS in MSSRF30^T^-plant interactions, as seen in T3SS-associated PGPR (53–56).

#### T6SS

**Fig 5:**
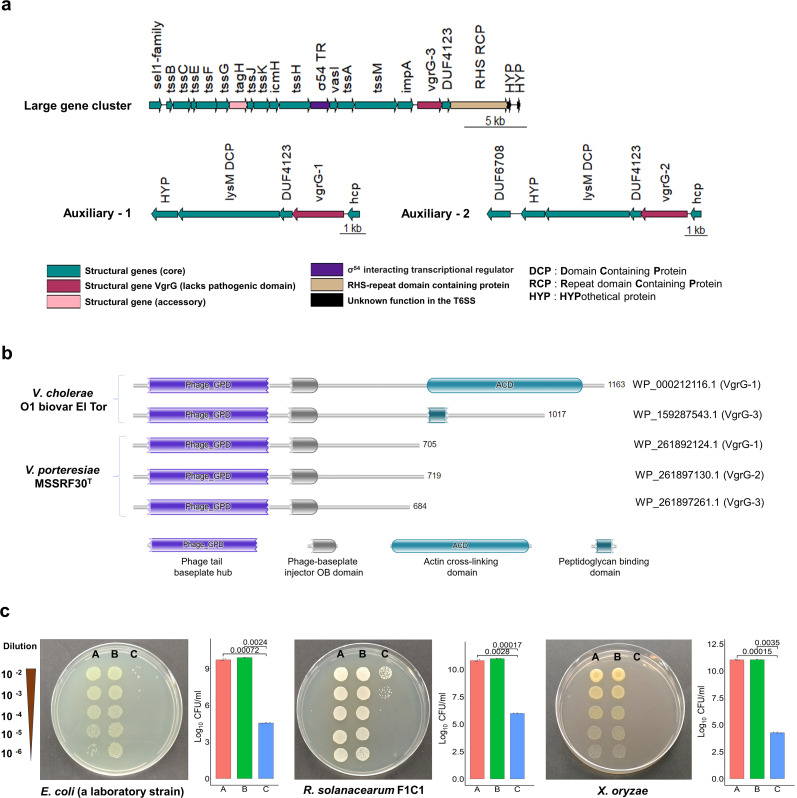
Type VI secretion system (T6SS) gene clusters of MSSRF30^T^. (**a**) The primary T6SS cluster contains 21 genes (large cluster), including all essential core components and two auxiliary clusters encode T6SS accessory proteins, likely contributing to specialized functions. (**b**) The domain architecture of predicted VgrG proteins in MSSRF30^T^ and *V. cholerae*. VgrG proteins are core components of the T6SS and contain conserved Phage_GPD, baseplate hub, and baseplate injector OB domains. Both strains retain the phage-derived components, but the additional actin cross-linking domain (ACD) and the peptidoglycan binding domain present in *V. cholerae* are not shared by *vgrG* proteins of MSSRF30ᵀ. Consequently, the latter are shorter in size, indicating that they may not be able to interfere with eukaryotic host actin dynamics and are specialized for alternative functions. (**c**) *In vitro* contact-dependent killing assay; MSSRF30ᵀ as predator and *E. coli*, *R. solanacearum*, and *X. oryzae* as prey were used. This experiment was performed under three conditions: (A) prey alone (negative control), (B) prey with *E. coli* DH5α (non-pathogenic competitor control), and (C) prey with MSSRF30ᵀ. The log_10_ 4 to log_10_ 5-fold reduction in recovery of prey bacteria is suggestive of the T6SS functional role in MSSRF30^T^ interbacterial competition.

MSSRF30^T^ genome encodes a complete set of T6SS-related genes organized as one large gene cluster and two auxiliary gene clusters, Aux-1 and Aux-2 (Fig. 5a). The large cluster encodes the main structural components of the T6SS, including a regulatory protein and two auxiliary clusters, each encoding a hemolysin-coregulated (Hcp) protein (Hcp1 or Hcp3) (Fig. 5a; Table S1g). In addition, all three clusters also contain genes coding for distinct valine-glycine repeat G proteins (VgrG-1, VgrG-2, and VgrG-3), T6SS-effector chaperones, and variable effector protein components. Notably, two T6SS core effector proteins, proline-alanine-alanine-arginine (PAAR) repeat-containing proteins (PAAR-1, WP_261892817, and PAAR-2, WP_261896979.1) and an Hcp2 (WP_26189022.1), were found outside the T6SS cluster. The main T6SS apparatus is very similar to that of *Vibrio cholerae* (data not shown). However, they lack polymorphic anti-eukaryotic toxins encoded in their C-terminal domains (Fig. 5b).

Like other T6SS-encoding vibrios, several T6SS effector proteins (T6Ep), including phospholipase effector Tle-1 like catalytic protein domain (WP_261892278.1), cell-wall degrading enzymes (WP_2618945731.1; WP_261895075.1), and lytic peptidase (WP-261893281.1), were identified outside of the T6SS cluster. These T6Ep are called cargo effectors, where they non-covalently interact with core proteins (Hcp, VgrG, or PAAR) and are thereby translocated into target cells upon T6SS contraction. These genomic findings indicate that T6SS, if expressed, can be functional. Studies have shown that T6SS plays an important role in interbacterial competition and host interaction (57, 58). To explore this possibility, an *in vitro* interbacterial growth competition assay was conducted. MSSRF30^T^ exhibits strong inhibitory effects, reducing the growth of *Escherichia coli*, *Xanthomonas oryzae*, and *Ralstonia solanacearum* cells by 4–5 log-fold (Fig. 5c). While these results are consistent with contact-dependent antagonism mechanisms such as T6SS, further experiments are required to confirm the specific genetic and molecular basis of this activity. Additionally, we found that T6SS-related genes were expressed in MSSRF30^T^ during its early root colonization (Table S2; Fig. S2), suggesting that T6SS in MSSRF30^T^ would play a significant role in host interactions and interbacterial competition, as seen in (57, 58).

In addition, the MSSRF30^T^ genome carries genes for the general secretion (Sec) pathway, twin-arginine translocation (TAT) pathway, and the Type I (T1SS) and II (T2SS) protein secretion systems (Table S1h), which are responsible for the translocation of proteins, both in folded and unfolded states, across the cytoplasmic membrane and into the extracellular environment.

### Plant-associated lifestyle

#### Motility, chemotaxis, and adhesion

Motility and chemotaxis are prerequisites for competitive root surface colonization and successful host establishment (59). MSSRF30^T^ encodes complete gene sets for a functional flagellar system (Table S1j) and contains three distinct chemosensory systems (clusters I, II, and III) and 62 chemoreceptors, a value higher than described for *V. cholerae* O1 biovar El Tor (45), *V. vulnificus* NBRC 15645 (46), *A. fischeri* ES114 (43), *V. harveyi* SB1 (31), and *V. parahaemolyticus* RIMD 2210633 (30). This higher number of chemoreceptors in MSSRF30^T^ potentially suggests its greater ability to sense and respond to the changing metabolic potential of its complex growth environment, i.e., plant rhizosphere . To support this view, we used an experimental setup comprising two phytajars separated by a permeable Whatman filter paper of 7–12 µm pore size (Fig. S3a). This result showed that a higher number of MSSRF30^T^ cells (1.0 ± 0.4 × 10^−8^ CFU/fresh gram of roots) were recovered from the roots than from the sand (1.5 ± 0.3 × 10^−4^ CFU/gram of sand), suggesting that MSSRF30^T^ could use these chemoreceptors to sense and navigate its movement in response to the metabolites secreted by the Pokkali root, leading to its efficient root colonization and establishment. Similar findings were reported in host-associated bacteria (59–63).

Additionally, several genes responsible for efficient host surface attachment were identified, including type IV pili, mannose-sensitive hemagglutinin (MSHA), PEP-CTERM surface anchoring with exopolysaccharides (Fig. S3b), and surface polysaccharides, were identified (Table S1k). Interestingly, the toxin co-regulated pilus (TCP), a major animal intestine colonization factor identified in *V. cholerae* O1 biovar EI Tor and *A. fischeri* ES114, was absent. Also, animal-associated *Vibrio* genomes lack PEP-CTERM surface anchoring with exopolysaccharides (data not shown). Notably, MSSRF30^T^ encodes a cellulose-binding protein belonging to the expansin EXLX1 family (WP_261897435.1), sharing 34.75–38.62% amino acid identity with EXLX1 homolog from plant-associated rhizobacteria, including beneficial (*Bacillus subtilis*) and phytopathogens (*Xanthomonas oryzae*, *Clavibacter michiganensis*, and *Ralstonia solanacearum*), which are involved in plant colonization (64–66). Importantly, *V. cholerae* O1 biovar EI Tor, *V. vulnificus* NBRC 15645, *A. fischeri* ES114, *V. harveyi* SB1, and *V. parahaemolyticus* RMID 2210633 genomes lack EXLX1 homolog. Additionally, we experimentally show that MSSRF30^T^ has a more binding affinity with rice roots (1.3 ± 0.2 × 10^−10^ CFU/g) than Caco-2 intestinal epithelial cells (1.7 ± 0.4 × 10^-6^ CFU/well) (Fig. S3c). These results indicate that MSSRF30^T^ has more evolved surface anchoring proteins mediating its firm host root attachment than intestinal epithelial cells. Further, our early root transcriptome data showed that several genes coding for motility, chemotaxis, and adhesions of MSSRF30^T^ are highly expressed (Table S2; Fig. S2), suggesting their potential involvement in plant interaction and successful colonization.

#### Sugars/dicarboxylate metabolism

MSSRF30^T^ can adhere, proliferate, and form a stable association with the Pokkali rice roots under a tested brackish condition (Fig. 2h through q), indicating that MSSRF30^T^ is well adapted to a plant-associated lifestyle. One such adaptation includes utilizing the major root-derived exometabolites, such as sugars and dicarboxylic acids, as found in other well-characterized PGPR (67). The genome of MSSRF30^T^ encodes complete metabolic enzymes necessary to carry out the Embden–Meyerhof–Parnas, the Entner–Doudoroff, the pentose-phosphate, tricarboxylic acid, and glyoxylate shunt pathways (Table S1m through p). Remarkably, MSSRF30^T^ carries multiple putative operons and individual genes encoding catabolic enzymes potentially involved in utilizing various plant-derived sugars and dicarboxylic acids (Table S1q). Furthermore, this genetic information was experimentally validated with a defined set of sugars and amino acids both individually and on a comparative scale (Fig. S4a, through h). More importantly, complete genetic systems encoding for arabinose, xylose, rhamnose, myo-inositol, gluconate, malonate, and glucarate metabolism were found to be absent in most of the well-studied host-associated *Vibrio* genomes (Fig. S5a), suggesting plant-specific metabolic adaptations of MSSRF30^T^. Studies have shown that PGPR-carrying catabolic genes for myo-inositol, malonate, rhamnose, and gluconate are robust root colonizers (68–70). Additional investigation predicted 523 diverse putative transport proteins in MSSRF30^T^, a value higher than the transporters described for other host-associated *Vibrio* genomes (Fig. S5b). Also, a comparative analysis of orthologous transporters among the assessed *Vibrio* genomes revealed that MSSRF30^T^ harbors 28 transporters distributed across 9 orthogroups that are unique to its genome (Fig. S6a and b). Further, our early root colonization transcriptome data reveals that several genes related to the transport and metabolism of plant-derived substrates, such as sugars and dicarboxylates, were significantly upregulated (Table S2). These results potentially reflect the specialized metabolic and uptake capacity of MSSRF30^T^ to utilize diverse plant-derived nutrients critical for its rhizosphere competence, colonization, and successful host establishment.

#### Plant polysaccharide utilization

According to the CAZy database (71), MSSRF30^T^ encodes 135 CAZymes (Table S1r), including 56 glycoside hydrolases (GHs), 32 glycosyltransferases (GTs), 20 carbohydrate-binding modules (CBMs), 17 polysaccharide lyases (PLs), and 10 carbohydrate esterases (CEs). The GHs belong to 26, and the PLs belong to six known families, markedly more than found in other assessed vibrios (Fig. S7), signifies the specialization for degrading plant cell wall polysaccharides.

**Fig 6:**
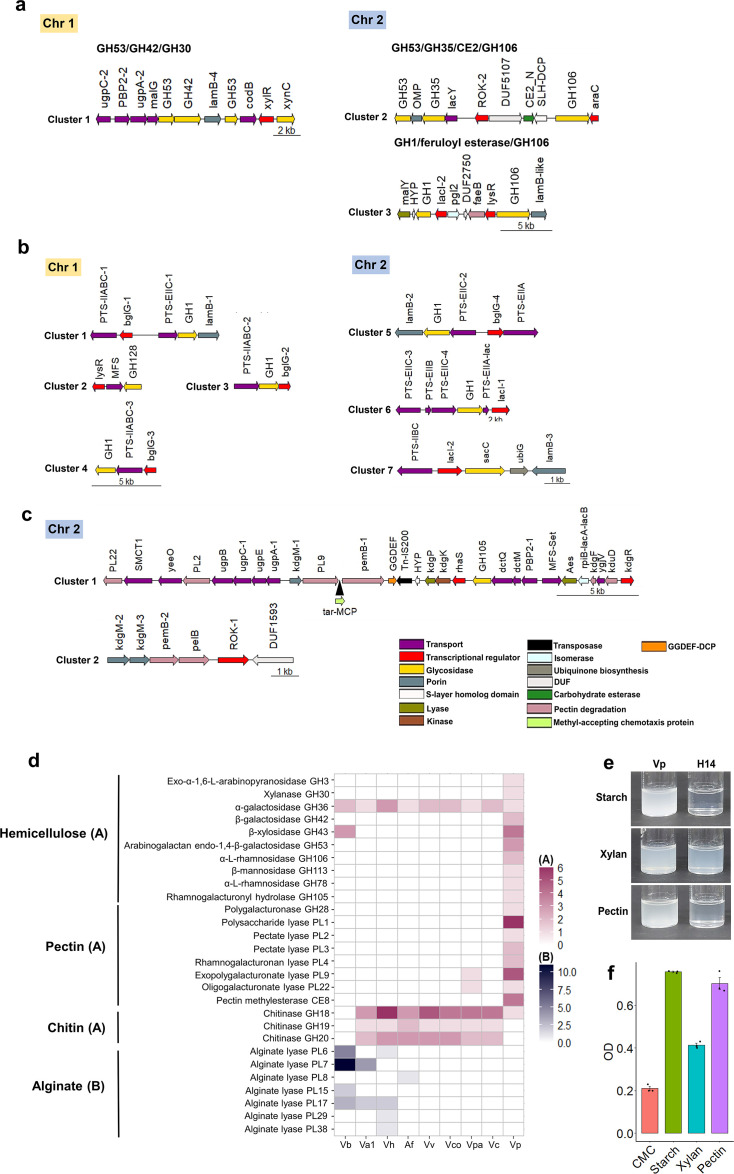
Plant polysaccharide-degrading CAZymes predicted in MSSRF30^T^. (**a**) Three putative gene clusters with multiple GH families linked with specific transport proteins and transcriptional regulators. (**b**) Seven putative gene clusters resembling the carbohydrate utilization loci. (**c**) Two potential gene clusters encoding putative CAZymes, specific transporters, porins, and transcriptional regulators that could digest pectin. (**d**) Heat map illustrating the various CAZymes identified in the genomes of MSSRF30^T^ and other host-associated vibrios, which are involved in the degradation of hemicellulose, pectin, chitin, and alginate. (**e and f**) The utilization of different plant polymers (starch, pectin, and xylan) by MSSRF30^T^ and *V. parahaemolyticus* strain H14.

The presence of three arabinogalactan endo-1,4-β-galactosidases (GH53), two β-xylosidases (GH43), two α-L-rhamnosidases (GH106), two β-galactosidases (GH42), one endo-1,5-α-L-arabinosidase (GH43), one xylanase (GH30), one exo-α-1,6-L-arabinopyranosidase (GH3), one α-galactosidase (GH36), and one β-mannosidase (GH113) indicates the capacity to degrade hemicellulose. Importantly, three putative gene clusters with multiple GH families linked with specific transport proteins and transcriptional regulators were predicted (Fig. 6a), suggesting that multiple GH families may work synergistically to digest complex hemicellulose, as shown in (72, 73).

Additionally, one cellulase (GH5/CBM2), five β-glucosidases (GH3), and nine β-glucosidases (GH1) indicate the capacity for cellulose degradation. Potential CAZymes that could depolymerize starch and β-glucan were also predicted (Table S1r). Notably, seven putative gene clusters resembling the carbohydrate utilization loci (74) were predicted (Fig. 6b). Each of these clusters encodes a GH family enzyme, one or more specific sugar transporters (PTS or MFS), and a specific transcriptional regulator (BglG or LacI) (Fig. 6b), indicating the potential to degrade and transport plant-derived carbohydrates. Further investigation identified two more potential gene clusters (one large and one small) carrying a collection of candidate genes, including CAZymes, specific transporters, porins, and transcriptional regulators that could digest pectin (Fig. 6c). Several pectin-degrading CAZymes, including one polygalacturonase (GH28), one α-L-rhamnosidase (GH78), multiple families of pectate lyases (5 PL1s, 2 PL3s, 2 PL4s, and 4 PL9s), and pectin methylesterases (2 CE8s), were predicated across the MSSRF30^T^ genome (Table S1r). Importantly, this genomic feature is rarely recognized in the *Vibrio* populations and is absent in all assessed *Vibrio* genomes, except that *V. parahaemolyticus* RIMD 2210633 has two PL families (PL9 and PL22) (Fig. 6d). Also, putative CAZymes responsible for alginate and chitin degradation were totally absent in MSSRF30^T^ (Fig. 6d). Although a GH18_chitinase-like subfamily protein was annotated in MSSRF30^T^, it lacks both catalytic and chitin-binding domains and, thus, may not be functional. Altogether, these genome results potentially reveal how profoundly the plant growth habitat has shaped the CAZyme inventory of MSSRF30^T^ during its evolution as plant-associated rhizobacteria. Also, multiple plant cell wall debranching CAZymes of MSSRF30^T^ might contribute to its rhizosphere fitness and efficient root colonization. In support of this later view, multiple GH families, GH1, GH2, GH32, GH43, and PL families of PL1 (1) and PL9 (4), were found to be upregulated during the early root colonization event of MSSRF30^T^ (Table S2; Fig. S2). Similar findings on the involvement of CAZymes in root colonization were reported in well-characterized diazotrophic endophytes, *Azoarcus* BH72 and *Azospirillum brasilense* Sp 245, and biocontrol PGPR, *Bacillus velezensis* GA1 (75–78).

Finally, our genomic predictions on plant cell wall–degrading CAZymes were experimentally validated to show positive growth of MSSRF30^T^ in xylan, pectin, and starch, but weak with carboxymethyl cellulose, and negative with chitin (Fig. 6e and f). Other potential CAZymes annotated in MSSRF30^T^ are summarized in Table S1r.

Additionally, key genome features related to plant association, such as quorum sensing, iron acquisition, and the lack of animal-associated virulence genes, are detailed in File S1.

### Conclusion

**Fig 7:**
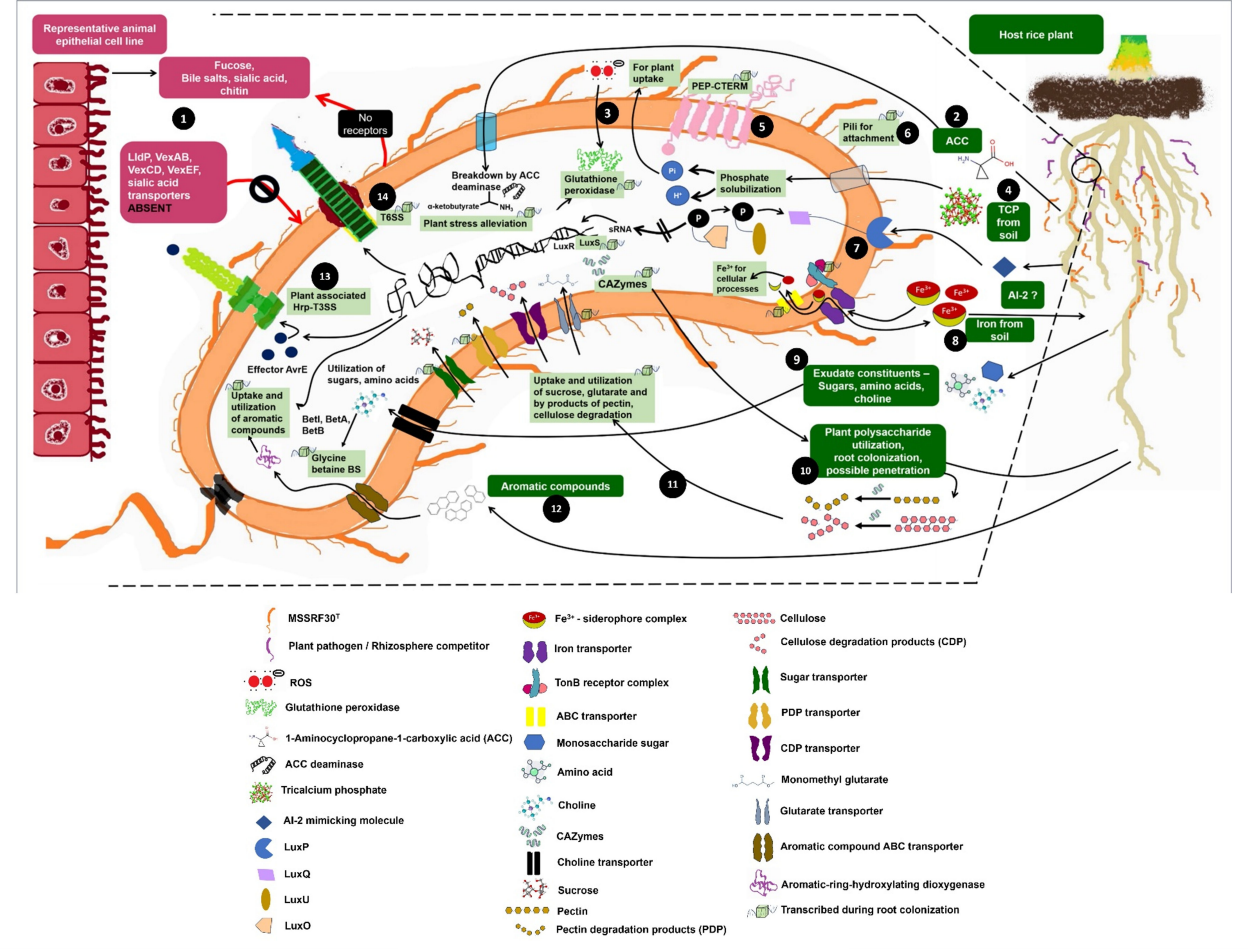
A graphical representation of the plant-associated lifestyle of MSSRF30^T^. (1) Absence of receptors for fucose, bile salts, sialic acid, and chitinase genes; lack of transporters LldP, VexAB/CD/EF, and sialic acid uptake systems, which are prerequisites for animal host association. (2) Plant-derived ACC is taken up and degraded by ACC deaminase, mitigating ethylene stress under salinity. (3) Plant-generated ROS is detoxified by glutathione peroxidase. (4) Tricalcium phosphate solubilized via a PQQ-independent pathway, releasing phosphate for plant uptake. (5) Presence of PEP-CTERM domains and (6) pili facilitate root surface attachment. (7) Chemosensing of AI-2-like plant signals triggers a phosphorelay quorum-sensing cascade like that observed in *V. harveyi* (79). (8) Siderophores produced chelate Fe; Fe-siderophore complexes are imported via TonB and ABC transporters, aiding both plant and bacterial Fe acquisition. (9) Root exudate sugars, amino acids, and choline are taken up and metabolized; choline is converted to glycine betaine for osmoprotection. (10) Controlled expression of CAZymes enables selective degradation of plant polysaccharides during colonization. (11) Uptake of polysaccharide degradation products such as sucrose and glucarate supports growth in the rhizosphere. (12) Aromatic compounds from the host are imported and catabolized via encoded genes. (13) Hrp-type T3SS is activated by plant signals, leading to efficient plant interaction. (14) T6SS might be involved in interbacterial competition and competitive rhizosphere colonization.

Using *V. porteresiae* MSSRF30^T^, a plant-associated rhizobacterium, along with brackish-associated salt-tolerant Pokkali rice as our research system, we demonstrate that MSSRF30^T^ possesses multiple plant growth-beneficial traits and significantly enhances plant growth in brackish environmental conditions for the first time. Additionally, root colonization studies further confirm that MSSRF30^T^ interacts and proliferates with high efficiency in rice roots. To uncover the genetic basis of these unique PGP traits, we performed a complete genome sequencing of MSSRF30^T^. This analysis identified several unique genes and genetic systems related to adaptation to a plant-associated lifestyle that had not been previously recognized in *Vibrio* species (Fig. 7), some of which are shared by vibrios isolated from similar environments (Table S3). Further, through *in planta* root transcriptome, we show the involvement of these gene systems in early root colonization and interaction. Interestingly, MSSRF30^T^ lacks key genome features critical for animal association, highlighting its genome plasticity. This study improves our understanding of *Vibrio* biology, especially their mutualistic relationship with brackish plants. Currently, we are beginning to investigate the complexities of these mechanisms of how *Vibrio* interacts with plants in brackish environments and their possible role in plant health and growth.

## MATERIALS AND METHODS

All bacterial strains used in this study are listed under “Strains”’ in the Supplemental text. A standardized plant nutrient medium was prepared, composed of half-strength Hoagland’s solution (wt/vol) with (PN-N^+^-20%NSW) or without (PN-N**^−^**-20%NSW) nitrogen and supplemented with 20% natural seawater (NSW) (vol/vol) to simulate a brackish environment. Seedlings were cultivated either in autoclavable plant tissue culture containers or in clerigel plates and transplanted to phyta jars or pots with artificial soil. For nitrogen fixation experiments, five-day-old seedlings were dip-inoculated for 1 hour in a cell suspension of MSSRF30^T^ (≥10^6^ cells) prepared in either PN-N**^+^**-20%NSW or PN-N**^−^**-20%NSW supplemented with 0.1% NH_4_Cl, resulting in two distinct sets of nitrogen-depleted and nitrogen-repleted plant growth conditions. The inoculated seedlings were then transplanted into segregated pots containing artificial soil. The qPCR assays were performed using MSSRF30^T^-specific primers for the *pyrH* and universal bacterial *nifH* primers for *nifH*. Assays for ACC deaminase production and differential growth in sugars and amino acids were performed using a modified M9 minimal medium. Growth in nitrogen-free media was confirmed using Jensen’s medium, and phosphate solubilization was assessed in Pikovskaya’s medium. Caco-2 cell line was obtained from the National Centre for Cell Science (NCCS), Pune, India. The *in vitro* assay was carried out with MSSRF30^T^ as the predator and a kanamcycin-resistant *E. coli* strain (a laboratory strain) and rifamycin-resistant phytopathogenic strains *Ralstonia solanacearum* F1C1 and *Xanthomonas oryzae* as prey organisms. The complete genome sequence of MSSRF30ᵀ was assembled by Genotypic Technology Pvt Ltd., Bangalore, India, using a hybrid approach integrating Illumina and Nanopore sequencing technologies. RNA sequencing was performed by Clevergene Biocorp Pvt. Ltd., Bangalore, India. The Illumina HiSeq plateform was used to sequence the RNA samples obtained from nitrogen-free Jensens broth, while Illumina NovaSeq 6000 platform was employed to sequence the RNA samples from early root colonization of MSSRF30^T^. A full description of methods is available in the Supplemental text.

## Data Availability

The assembled genome has been deposited in the NCBI GenBank database under accession number PRJNA1034238. The RNA-Seq data generated from gene expression studies under nitrogen-free conditions and early root colonization were deposited to the NCBI Sequence Read Archive database under accession numbers PRJNA1117903 and PRJNA1121645, respectively.

## SUPPLEMENTAL MATERIAL

File S1 msystems.00910-25-s0001.pdf Plant-associated genome features in MSSRF30^T^ with reference to quorum sensing, iron acquisition, and lack of animal-associated virulence factors.

Supplemental text msystems.00910-25-s0002.pdf Complete methods.

Supplemental material msystems.00910-25-s0003.pdf Figures S1 to S7.

Table S1 msystems.00910-25-s0004.pdf MSSRF30^T^ genome features.

Table S2 msystems.00910-25-s0005.xlsx Upregulated genes in MSSRF30^T^ during early root colonization.

Table S3 msystems.00910-25-s0006.xlsx Plant-asscociated genes predicted in other *Vibrio* genomes.

Movie S1 msystems.00910-25-s0007.mp4 Preferential colonization of MSSRF30^T^ at the lateral root junction of an eight day old Pokkali rice seedling under a brackish condition.

ASM does not own the copyrights to Supplemental Material that may be linked to, or accessed through, an article. The authors have granted ASM a non-exclusive, world-wide license to publish the Supplemental Material files. Please contact the corresponding author directly for reuse.
