# Supplementary material for: Wild rice-associated Vibrio promotes plant growth and exhibits genomic and phenotypic plasticity for plant adaptations
Source: mSystems. 2025 Oct 27;10(11):e00910-25. doi: 10.1128/msystems.00910-25 (PMC12625758; doi:10.1128/msystems.00910-25)
Supplement: File S1 — Plant-associated genome features in MSSRF30T with reference to quorum sensing, iron acquisition, and lack of animal-associated virulence factors. [file msystems.00910-25-s0001.pdf]

Supplementary main text file S1.

Quorum sensing: The MSSRF30^T genome encodes a functional autoinducer-2 (AI-2) quorum-sensing system (Table S1i). This AI-2 system may enable them to communicate within and between different species and modulate the expression of multiple genes in response to cell population density and the metabolic potential of an environment, as seen in host-associated vibrios (1).

Iron acquisition: MSSRF30^T is negative for siderophore production, and its genome encodes genes for enterobactin biosynthesis and secretion (Table S1l). Notably, MSSRF30^T encodes a higher proportion of genes related to iron transport and has more TonB iron transporters (14) compared to *V. cholerae* O1 biovar El Tor (6), *A. fischeri* ES114 (8), and *V. parahaemolyticus* RIMD 2210633 (10). Also, specific iron-binding receptors for catecholate- and hydroxamate-type siderophores, enterobactin, ferric citrate, heme, and hemoglobin, and their cognate transporter systems were identified. These results suggest that MSSRF30^T is highly adapted to scavenge iron through siderophores produced by other resident rhizobacteria or by the host, thereby enhancing its growth and rhizosphere competence under iron-limiting conditions (2).

Lack of animal-associated virulence genes

Virulence factors: Genes associated with the pathogenicity of *Vibrio cholerae* O1 biovar El Tor str, including CTXϕ, a chromosome-integrated bacteriophage that carries c*txAB* (encoding A and B subunits of cholera toxin, CT), *cep* (encoding core-encoded pilin), *orfU* (encoding a product of unknown function), and the repetitive sequence (RS) elements (*rstRABC*) were absent in MSSRF30^T. However, two genes, *zot* (encoding zonula occludens toxin) and DUF2523 family protein (homology of accessory enterotoxin), were identified as part of the phage element in MSSRF30^T, but their functions are yet to be determined. In addition, CTXϕ-linked RTX (repeats in toxin) toxin (*rtxA*) and its transcriptional activator (*rtxC*) and ABC transporter system (*rtxBD*), crucial for cytotoxicity (3), and complete toxin co-regulated pilus (TCP) apparatus (*tcpABCDEFGHIJNQRST*) as well as ToxT and TcpP (two major virulence transcriptional activators), essential for intestinal colonization and infection (4), were found absent. Additionally, key genes that enhance virulence, including the lysine/cadaverine antiporter (*cadB*), lysine decarboxylase (*cadA*) and its activator (*cadC*), which are essential for

acid tolerance (5), and outer membrane porin (*ompU*), associated with bile resistance (6), were not identified. Further, MSSRF30^T lacks other major virulence factors such as thermostable direct hemolysin (*tdH*) and its homology *trH* (encoding *tdH*-related hemolysin) of *V. parahaemolyticus* (7) and phospholipase A2 (*plpA*) and cytolysin/ hemolysin (*vvhA*) of *V. vulnificus* (8). Strikingly, MSSRF30^T encodes a complete phytopathogen hrp type-T3SS (Fig. 4a) but lacks the canonical animal pathogen type-T3SS. Like many other non-pathogenic vibrios, MSSRF30^T encodes ToxRS, a transmembrane regulatory system, and an increase in *toxS* gene expression ($\log_2$ FC = 1.90) was observed during the early pokkali rice root colonization of MSSRF30^T (Table S2), suggesting these regulators may play an important role in plant interactions.

References

1. Miller MB, Bassler BL. 2001. Quorum sensing in bacteria. *Annu Rev Microbiol* 55:165–199
2. Jiao X, Takishita Y, Zhou G, Smith DL. 2021. Plant associated rhizobacteria for biocontrol and plant growth enhancement. *Front Plant Sci* 12:634796.
3. Son MS, Megli CJ, Kovacikova G, Qadri F, Taylor RK. 2011. Characterization of *Vibrio cholerae* O1 El Tor biotype variant clinical isolates from Bangladesh and Haiti, including a molecular genetic analysis of virulence genes. *J Clin Microbiol* 49:3739–3749.
4. Krukonis ES, Yu RR, Dirita VJ. 2000. The *Vibrio cholerae* ToxR/TcpP/ToxT virulence cascade: distinct roles for two membrane-localized transcriptional activators on a single promoter. *Mol Microbiol* 38:67–84.
5. Ante VM, Bina XR, Bina JE. 2015. The LysR-type regulator LeuO regulates the acid tolerance response in *Vibrio cholerae*. *Microbiology* 161:2434–2443.
6. Wibbenmeyer JA, Provenzano D, Landry CF, Klose KE, Delcour AH. 2002. *Vibrio cholerae* OmpU and OmpT porins are differentially affected by bile. *Infect Immun* 70:121–126.
7. Raghunath P. 2015. Roles of thermostable direct hemolysin (TDH) and TDH-related hemolysin (TRH) in *Vibrio parahaemolyticus*. *Front Microbiol* 5:805.
8. Kim BS. 2020. Spatiotemporal regulation of *Vibrio* exotoxins by HlyU and other transcriptional regulators. *Toxins* 12:544.
