# Supplementary material for: Wild rice-associated Vibrio promotes plant growth and exhibits genomic and phenotypic plasticity for plant adaptations
Source: mSystems. 2025 Oct 27;10(11):e00910-25. doi: 10.1128/msystems.00910-25 (PMC12625758; doi:10.1128/msystems.00910-25)
Supplement: Supplemental text — Complete methods. [file msystems.00910-25-s0002.pdf]

Methods

Strains

Vibrio porteresiae (type strain MSSRF30^T = DSM 19223^T) was obtained from the Deutsche Sammlung von Mikroorganismen und Zellkulturen (DSM), along with *Vibrio mangrovi* (type strain MSSRF38^T = DSM 19640^T), *Vibrio ruber* (MSSRF10 = DSM 18600), *Vibrio plantisponsor* (type strain MSSRF60^T = DSM 21026^T), and the two strains of *Vibrio rhizosphaerae*, MSSRF3^T (type strain MSSRF3^T = DSM 18581^T) and MSSRF7 (strain MSSRF7 = DSM 18601). For the fluorescence imaging of root-bacteria interactions, MSSRF30^T was transformed with a GFP-expressing vector pKV111 harbouring a gene for chloramphenicol resistance through conjugation using *E.coli* CC118 λpir as the donor strain. A *Burkholderia* sp. strain BmKn7 isolated from the roots of another in-land rice variety (Aishwarya) was used as the positive control for the quantification of ACC deaminase activity and plate assays of phosphate solubilization. Additionally, *Vibrio parahaemolyticus* strain H14 was employed as a positive control for Caco-2 cell adhesion studies, the PGPR isolate *Pseudomonas putida* UW4 was included as a positive control for the comparative studies on plant growth, and *Azoarcus* strain L1K30 was used as a positive control for endophytic colonization studies.

Media and substrates for plant experiments

Seedling experiments were carried out in sterile, clear, vented, autoclavable plant tissue culture containers (360 ml) with filter sheets manually fitted into them for a duration of 10 days. Seedlings were alternatively grown in pots with artificial soil (a 2:1 blend of vermiculite and quartz sand) for 20-day experiments. Root colonization was assessed in autoclavable square phyta jars (500 ml) using a hydroponic system. All gnotobiotic plant experiments were conducted using a standardized plant nutrient medium composed of half-strength Hoagland solution (w/v) with nitrogen (HiMedia, TS1094, abbreviated as PN-N$^+$- 20%NSW) or without nitrogen (HiMedia, TS1117, abbreviated as PN-N$^-$-20%NSW) supplemented with 20% natural seawater (v/v NSW) to simulate a brackish environment. The seawater concentration was optimized to support the pokkali rice and bacterial growth.

Plant germination, transplantation, and cultivation

The pokkali seeds were surface sterilized by rinsing them in a 6% solution of sodium dichloroisocyanurate dihydrate (Sigma-Aldrich, 218928) and 0.1% Tween 80 for 20 min, which was followed by immersing them in sterile distilled water for overnight imbibition. Subsequently, the seeds were aseptically transferred to 1.5% Clerigel (HiMedia, PCT0903) for the following 6 days for germination. The sprouted seeds were incubated in a growth chamber (BINDER, KBW 720) with a photoperiod of 11 hours light/13 hours dark, at 30°C, 50% relative humidity, a thermal flux of 38 W/m^2, and illuminance of 13,000 lx. Seedlings exhibiting uniform growth were transplanted to a hydroponic system, maintained in polypropylene phyta jars containing approximately 300 ml of seawater-infused half-strength Hoagland solution, or into pots with artificial soil, semi-submerged in

trays filled with the same plant nutrient solution. The nutrient solution in the trays was replenished every 2 days. For the pot experiments, plants were maintained in a shaded net house to simulate a greenhouse-like environment.

Root colonization and plant growth promotion studies

For root growth promotion studies, 3-day-old seedlings were incubated for 1 hour in a cell suspension of MSSRF30^T ($\geq 10^6$ cells) prepared in 20% natural seawater-supplemented half-strength Hoagland solution, after which they were transplanted into either autoclavable vented containers or pots with artificial soil and cultivated as described above, for 10 or 20 days, respectively.

For root colonization studies, sterile 7-day-old seedlings were transplanted into phyta jars and inoculated with a 10^4 CFU/mL cell suspension of MSSRF30^T or GFP-tagged MSSRF30^T, suspended in the same nutrient solution as that in the jars. The nutrient solution was replaced with a fresh nutrient solution every 2 days throughout the experiment. Roots were excised from each plant every fourth day and thoroughly washed using a vortexer. Six biological replicates were collected on each sampling day for a period of 12 days. The roots were dried on sterile Whatman filter paper to remove surface moisture, then crushed in microfuge tubes using a sterile micropestle. The crushed root material was serially diluted 10-fold, and aliquots were spotted onto growth media plates to calculate the CFU/g of fresh root weight. In parallel, total DNA was isolated from segregated sets of bacteria-colonized roots every fourth day. Extraction was done by pulverizing the excised roots in liquid nitrogen, followed by extraction using the QIAGEN DNeasy Plant kit. Quantitative PCR (qPCR) was performed on the normalized DNA samples using MSSRF30^T-specific primers targeting the UMP kinase (*pyrH*) gene; Forward (CAACCGTTCTTGACCGCATG) and Reverse (CCACCGATGACAACACCAAC). The cycling conditions included an initial 4-minute incubation at 95°C, followed by 30 cycles of 95°C for 1 minute, 51°C for 30 seconds, and 72°C for 20 seconds, with a final extension at 72°C for 5 minutes. Amplification specificity was confirmed by melting curve analysis of the PCR products. Absolute quantification of the cycle threshold (Ct) values was performed to estimate the copy number of *pyrH* in each sample. The GFP images were captured using the Carl Zeiss LSM 980 inverted confocal microscope, and the movie using the epifluorescent Carl Zeiss Axio Imager M2, with GFP filter cube (Ex: 470/40; Em: 525/50).

For the chemotaxis experiment, the permeable barrier (Whatman filter paper with 7-12 micron pore size) was placed beneath the quartz sand, separating the plant nutrient solution containing cells of MSSRF30^T from the host plants grown in the sand. After 3 days of incubation, the CFU/g recovery of MSSRF30^T was measured from the roots and 1g of sand from the non-host negative control. The experiment was performed independently in duplicate.

In planta nitrogen fixation studies

Seedlings were cultivated as explained in the main text. Plants were grown in the shaded net house as described in prior experiments. At 20 days post-inoculation (DPI), plants were harvested to assess shoot health, nitrogen content, and confirm differential expression of the nitrogenase gene (*nifH*). Total nitrogen content in the shoots was determined using a Kjeldahl Automatic Nitrogen Protein Analyzer (KjelTRON, Tulin Equipment, India), which integrates all 3 steps of the Kjeldahl process into a single system. Briefly, samples were digested using concentrated sulphuric acid, followed by distillation with 50% sodium hydroxide. The released ammonia was condensed in 4% boric acid and titrated against 0.1N hydrochloric acid to obtain the amount of ammonia released. This was then used to calculate the total nitrogen content using the standard Kjeldahl equation. For the quantification of the nitrogenase gene, liquid nitrogen was used to pulverize plant roots, and total RNA was extracted using the QIAGEN RNeasy Mini kit (74104). Genomic DNA was removed using the DNase I, RNase-free kit (Thermo Scientific™, 1U/µL, EN0521) according to the manufacturer's instructions. The NEB ProtoScript® II First Strand cDNA synthesis kit (E6560S) was used to synthesize cDNA. The qPCR assay was performed using the *nifH* gene-specific primers; 19F (GCIWTYTAYGGIAARGGIGG) and 388R (AAICCRCCRCAIACIACRTC). The cycling conditions were as follows: a 4-minute incubation at 95°C, 30 cycles of 95°C for 1 minute, 54°C for 30 seconds, and 72°C for 20 seconds, followed by a final 5-minute incubation at 72°C. The results were analyzed using the 2-ΔΔCt method for relative quantification of *nifH* expression in nitrogen-free and NH_4Cl-supplemented plants.

In vitro assays for plant growth-promoting traits

To confirm growth in ACC, a modified M9 minimal medium was prepared containing sodium phosphate dibasic (0.678 g/L), potassium dihydrogen phosphate (0.3 g/L), potassium phosphate dibasic (0.8 g/L), magnesium sulphate heptahydrate (0.5 g/L), calcium chloride dihydrate (1 g/L), sodium chloride (20 g/L), fructose (5 g/L) and 3 mM of ACC or ammonium chloride. OD_{600} measurements were taken every 24 hours up to 72 hours post-inoculation (HPI). ACC deaminase enzyme activity was assessed at 48 HPI (hours post inoculation) for $MSSRF30^T$ and $MSSRF38^T$ and at 72 HPI for the other strains, following the protocol outlined by Krishnan et al. (1). For the ACC growth assay using UW4, the same medium composition was utilized, but instead of 2% NaCl, 0.5% NaCl was used. Additionally, fructose was substituted with glucose at a concentration of 5 g/L. The growth in ACC was also assessed in a modified M9 minimal medium that contained 20% natural seawater.

To culture $MSSRF30^T$ in nitrogen-free broth or semi-solid media, Jensen's broth (granulated, HiMedia, GM973), prepared at half-strength and supplemented with 1.95% NaCl to achieve a final NaCl concentration of 2%, was used. For semi-solid conditions, 0.3% purified agar was used. For phosphate solubilization, Pikovskaya's medium (HiMedia, M520) supplemented with 2% NaCl was used for $MSSRF30^T$. For BmKn7, Pikovskaya's medium was supplemented with 0.5% NaCl. For the estimation

of gluconic acid, MSSRF30^T was inoculated in Pikovskaya's broth and incubated at 30°C in the shaker. Samples were collected from the inoculated broth at 4-hour intervals up to 12 hours and centrifuged to obtain the supernatant for HPLC analysis. The mobile phase used was 0.01N sulphuric acid and a Rezex ROA-Organic Acid H+ (8%) as the organic acid analytical column (Phenomenex, Product no. 00G-0138-E0). Standard solutions of gluconic acid (D-gluconic acid sodium salt, Sigma-Aldrich, G9005) were prepared at concentrations of 2, 4, 6, 8, and 10 mg/ml. The resulting chromatograms were extrapolated as the peak area against the concentration in mg/ml. Phosphate solubilization was also assessed by measuring the release of inorganic phosphate (Pi). A modified version of Pikovskaya's broth was prepared, replacing the tricalcium phosphate with monosodium dihydrogen phosphate (NaH_2PO_4). Standard solutions of NaH_2PO_4 were prepared in a concentration range of 0 to 275 nmol/µl. The supernatant from the inoculated broth was filtered, and Pi release was quantified using the ammonium molybdate–ascorbic acid method (2,3). A linear regression model was constructed in RStudio to predict gluconic acid concentrations and Pi release. The concentrations of gluconic acid or the amount of Pi released in the supernatant of each sample were calculated using this model.

For zinc solubilization, modified M9 minimal medium was supplemented with 0.1% zinc oxide, and the zone of clearance was recorded three days after spotting the culture onto the medium plate.

Growth in sugars and plant polysaccharides as sole carbon sources

A single colony of MSSRF30^T grown on Zobell Marine Agar 2216 (HiMedia, M384) was washed and resuspended in a 0.85% saline solution. The suspended cells were inoculated into a minimal medium containing the respective carbon source. The modified M9 minimal medium served as the base medium. The sugars used in the experiment included cellobiose, fructose, arabinose, mannose, maltose, lactose, xylose, galactose, sucrose, glucose, rhamnose, trehalose, and raffinose. The dicarboxylic acids tested were malic acid and salicylic acid, while mannitol was the sole sugar alcohol included. The amino acids incorporated were tryptophan, histidine, arginine, proline, serine, lysine, glycine, alanine, valine, threonine, asparagine, glutamine, methionine, isoleucine, leucine, phenylalanine, putrescine, and cysteine. Each sugar or amino acid was added to the base medium at a concentration of 5 g/L in a 48-well microtiter plate and incubated at 30°C. Optical density (OD_{600}) values were recorded for all aliquots after 48 hours of incubation. Growth on different polysaccharides, such as starch, xylan, pectin, carboxymethyl cellulose, and chitin, was assessed in 30 ml flasks containing modified M9 base medium supplemented with respective polysaccharides at 5 g/L. OD_{600} values were recorded after 72 hours of incubation.

Caco-2 cell adhesion studies

Caco-2 obtained from the National Centre for Cell Science (NCCS), Pune, India, was cultured and maintained in Dulbecco's Modified Eagle Medium (DMEM; Sigma) supplemented with 10% fetal

bovine serum (FBS; Himedia) and 1% antibiotic-antimycotic solution (10,000 U/mL penicillin, 10 mg/mL streptomycin, and 25 µg/mL amphotericin B in 0.9% saline; Himedia). The cell line was maintained in a humidified incubator with 5% CO_2 for 6 to 8 days at 37°C. To assess bacterial adhesion, 2×10^5 Caco-2 cells were seeded into 6-well plates and allowed to reach confluence. Cell suspensions of MSSRF30^T and H14 were pre-washed with PBS and applied to the cell monolayers in triplicate at an approximate multiplicity of infection (MOI) of 10:1 and incubated at 37 °C for 2 hours. The CFU/ml of the original inoculum was determined using quantitative spot plating. Following incubation, the wells were washed thrice to remove free-floating bacteria. The monolayers were then lysed using 0.1% Triton X-100 (Sigma-Aldrich) to release adherent bacteria. Lysates were serially diluted and plated on ZMA, followed by overnight incubation at 30°C to determine colony-forming units (CFU/ml). Adhesion efficiency was calculated by expressing the number of adhered bacteria as a percentage of the bacterial count in the initial inoculum. The procedure was adapted from the method developed by Elsinghorst and Kopecko (4) and modified by Prabhakaran et al. (5), with minor modifications made in the current study. The experiment was performed thrice for statistical reliability.

In vitro predator-prey contact-dependent killing assay

This experiment was performed as described previously (6). Briefly, the assay was conducted on nutrient agar media by manually preparing a mixture of predator and prey cells in a 1:1 ratio, followed by a 12-hour incubation. The spotted mixture was then serially diluted and spotted on the respective growth media containing antibiotics for the selection of prey bacteria. Prey colonies were enumerated from the dilution spots after 72 hours of incubation at 30°C.

Genome sequencing and analysis

Functional annotation was performed using the NCBI Prokaryotic Genome Annotation Pipeline (PGAP), and annotated files and tables from the submission were utilized for downstream analyses. Prophage regions were identified using PHASTER (7), tRNAs were predicted with tRNAscan-SE v2.0 (8), and integrons were detected using ISEScan v1.7.2.3 (9). Orthology assignments were done using eggNOG-mapper v2 (10). The whole-genome BLASTp tool from the Bacterial and Viral Bioinformatics Resource Center (11) was used to identify orthologous genes associated with nitrogen fixation, secretion systems, and virulence and validated through reciprocal BLAST analysis. For comparison of *nif* regions, the genomes of model organisms—*Stutzerimonas stutzeri* A1501 (formerly *Pseudomonas stutzeri* A1501), *Azotobacter vinelandii* DJ, *Azoarcus olearius* BH72, *Azospirillum brasilense* Sp7^T, and *Herbaspirillum seropedicae* SmR1 were retrieved from NCBI. Domain differences in the T6SS spike protein VgrG were identified and visualized using the HMMER web server v2.39.0 (12). Genes related to chemotaxis, motility, adhesion, sugar, and plant polysaccharide utilization were identified through manual curation, while specific genes encoding glycosyl hydrolases, pectate lyases, chitinases, and alginate lyases were identified and classified using the CAZy database

(13). MSSRF30^T-specific transporters were identified using OrthoFinder version 2.5.5 (14). KEGG analysis was employed to categorize genes involved in metabolic pathways. All BLASTp analyses were performed using a threshold identity value of 50% (0.5) or higher, with a coverage range of 80% to 100%.

The operons and other gene clusters displayed were organized based on size and orientation and visualized using the genoPlotR package in R v0.8.11 (15). Genes are color-coded by function as indicated in each legend. Arrows indicate transcriptional orientation. Scale bar representations are as shown.

The genomes of MSSRF38^T, MSSRF60^T, and MSSRF7, submitted under PRJNA1034771, were used to identify conserved plant-associated genes.

Transcriptome profiling and differential gene expression analysis (DGEA)

For RNA-Seq analysis of gene expression under nitrogen-free conditions, MSSRF30^T was cultured in Jensen's medium containing 2% NaCl with or without supplementation of 0.1% NH_4Cl. Total RNA was isolated 9 hours post-inoculation.

For the early root colonization transcriptome study, total RNA was extracted from roots at 3 DPI, followed by DNA removal as previously described. RNA from cells in the planktonic state, cultured in modified M9 broth containing 0.5% fructose, was used as the control group. Both analyses were performed with a triplicate of each condition.

The quality of the raw reads was assessed using the graphical user interface version of FastQC v0.12.1 (16), which confirmed that all reads had a base quality score above 28. Adapter sequences were removed, and reads shorter than 28 base pairs were filtered using Trimmomatic v0.39 (17). The processed reads were aligned to the MSSRF30^T genome using HISAT2 v2.2.1, and the resulting BAM files were used as input for feature Counts v2.0.6 (18) to generate raw gene-level counts. These raw counts were log-transformed, and significantly differentially expressed genes were identified using DESeq2() in RStudio (19). Genes with a log2 fold change (log2FC) threshold of ≤ 0.01 were considered significant for the nitrogen-free DGEA, while a log2FC threshold of ≤ 0.05 was applied for the early root colonization DGEA.

Statistical analysis

R package ggplot2 v3.5.1 (20) was used to perform statistical tests and to generate all the graphs except for the Caco-2 adhesion index, which was plotted using GraphPad Prism v10.4.0 (GraphPad Software, Boston, MA, USA, https://www.graphpad.com). UpSetR v1.4.0 (21) was used to generate the upset

plot to show intersecting orthogroups of transporters. Data are presented as mean ± SE, and p-values were calculated using Student's t-test, one-way ANOVA as indicated in the figure legends.

References

1. Krishnan R, Lang E, Midha S, Patil PB, Rameshkumar N. 2018. Isolation and characterization of a novel 1-aminocyclopropane-1-carboxylate (ACC) deaminase producing plant growth promoting marine Gammaproteobacteria from crops grown in brackish environments. Proposal for *Pokkaliibacter plantistimulans* gen. nov., sp. nov., *Balneatrichaceae* fam. nov. in the order *Oceanospirillales* and an emended description of the genus *Balneatrix*. *Syst Appl Microbiol* 41:570–580.
2. Knowles V, Plaxton W. 2013. Quantification of total and soluble inorganic phosphate. *Bio-protocol* 3:e890.
3. Hurley BA, Tran HT, Marty NJ, Park J, Snedden WA, Mullen RT, Plaxton WC. 2010. The dual-targeted purple acid phosphatase isozyme AtPAP26 is essential for efficient acclimation of *Arabidopsis* to nutritional phosphate deprivation. *Plant Physiol* 153:1112–1122.
4. Elsinghorst EA, Kopecko DJ. 1992. Molecular cloning of epithelial cell invasion determinants from enterotoxigenic *Escherichia coli*. *Infect Immun* 60:2409–2417.
5. Meparambu Prabhakaran D, Ramamurthy T, Thomas S. 2020. Genetic and virulence characterisation of *Vibrio parahaemolyticus* isolated from Indian coast. *BMC Microbiol* 20:1–14.
6. Salomon D, Gonzalez H, Updegraff BL, Orth K.2013. *Vibrio parahaemolyticus* type VI secretion system 1 is activated in marine conditions to target bacteria, and is differentially regulated from system 2. *PLoS One* 8:e61086.
7. Arndt D, Grant JR, Marcu A, Sajed T, Pon A, Liang Y, Wishart DS. 2016. PHASTER: a better, faster version of the PHAST phage search tool. *Nucleic Acids Res* 44:W16–21.
8. Chan PP, Lin BY, Mak AJ, Lowe TM. 2021. tRNAscan-SE 2.0: improved detection and functional classification of transfer RNA genes. *Nucleic Acids Res* 49:9077–9096.
9. Xie Z, Tang H. 2017. ISEScan: automated identification of insertion sequence elements in prokaryotic genomes. *Bioinformatics* 33:3340–3347.
10. Cantalapiedra CP, Hernández-Plaza A, Letunic I, Bork P, Huerta-Cepas J. 2021. eggNOG-mapper v2: functional annotation, orthology assignments, and domain prediction at the metagenomic scale. *Mol Biol Evol* 38:5825–5829.
11. Olson RD, Assaf R, Brettin T, Conrad N, Cucinell C, Davis JJ, Dempsey DM, Dickerman A, Dietrich EM, Kenyon RW, Kuscuoglu M. 2023. Introducing the bacterial and viral bioinformatics resource center (BV-BRC): a resource combining PATRIC, IRD and ViPR. *Nucleic Acids Res* 51:D678–689.
12. Potter SC, Luciani A, Eddy SR, Park Y, Lopez R, Finn RD. 2018. HMMER web server: 2018 update. *Nucleic Acids Res* 46:W200–204.

13. Lombard V, Golaconda Ramulu H, Drula E, Coutinho PM, Henrissat B. 2014. The carbohydrate-active enzymes database (CAZy) in 2013. *Nucleic Acids Res* 42:D490–D495.
14. Emms DM, Kelly S, 2019. OrthoFinder: phylogenetic orthology inference for comparative genomics. *Genome Biol* 20:1-14.
15. Guy L, Roat Kultima J, Andersson SG. 2010. genoPlotR: comparative gene and genome visualization in R. *Bioinformatics* 26:2334–2335.
16. Andrews, S., 2010. FastQC: a quality control tool for high throughput sequence data. Available online at: https://www.bioinformatics.babraham.ac.uk/projects/fastqc/
17. Bolger AM, Lohse M, Usadel B. 2014. Trimmomatic: a flexible trimmer for Illumina sequence data. *Bioinformatics* 30:2114–2120.
18. Liao Y, Smyth GK, Shi W. 2014. featureCounts: an efficient general purpose program for assigning sequence reads to genomic features. *Bioinformatics* 30:923–930.
19. Love MI, Huber W, Anders S. 2014. Moderated estimation of fold change and dispersion for RNA-seq data with DESeq2. *Genome Biol* 15:550.
20. Wickham H, 2009. Ggplot2: elegant graphics for data analysis, 2nd edition, Springer, New York.
21. Conway JR, Lex A, Gehlenborg N, 2017. UpSetR: an R package for the visualization of intersecting sets and their properties. *Bioinformatics* 33:2938-2940.
