# Supplementary material for: Wild rice-associated Vibrio promotes plant growth and exhibits genomic and phenotypic plasticity for plant adaptations
Source: mSystems. 2025 Oct 27;10(11):e00910-25. doi: 10.1128/msystems.00910-25 (PMC12625758; doi:10.1128/msystems.00910-25)
Supplement: Supplemental material — Figures S1 to S7. [file msystems.00910-25-s0003.pdf]

Supplementary Figures

a

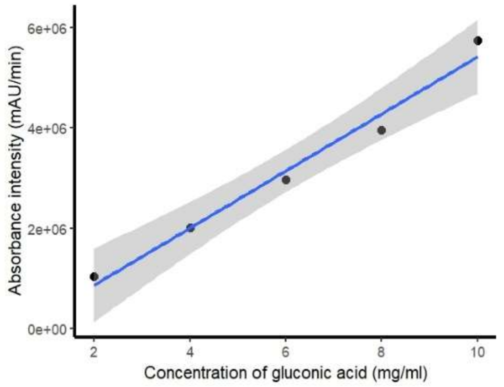

b

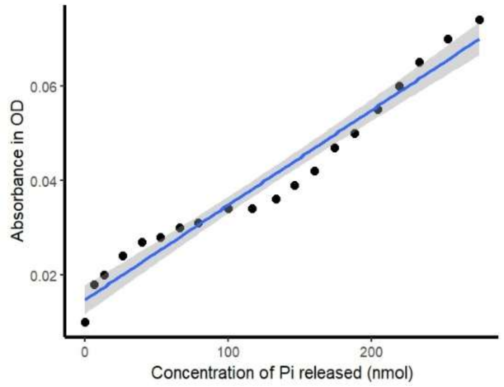

Hours post-inoculation	Absorbance intensity (mAU/min)	Predicted concentration
4	520196	1.49
4	517945	1.48
4	519698	1.49
8	1155487	2.58
8	1148452	2.57
8	1136738	2.55
12	2100546	4.21
12	2009664	4.05
12	2101341	4.21

Condition	Optical density	Pi release (nmol)
Control	0.040	125.72
Control	0.041	130.49
Control	0,043	135.27
MSSRF30^T	0.069	264.22
MSSRF30^T	0.067	254.67
MSSRF30^T	0.063	235.57

c

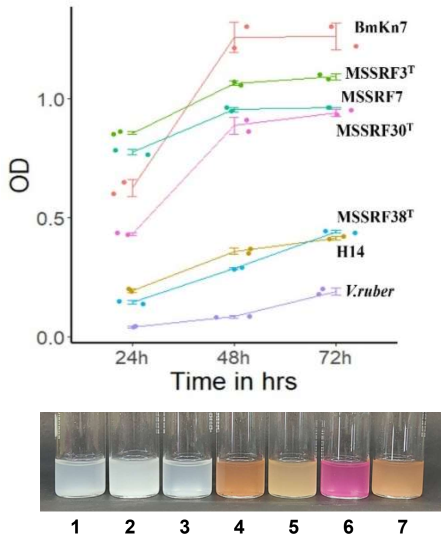

d

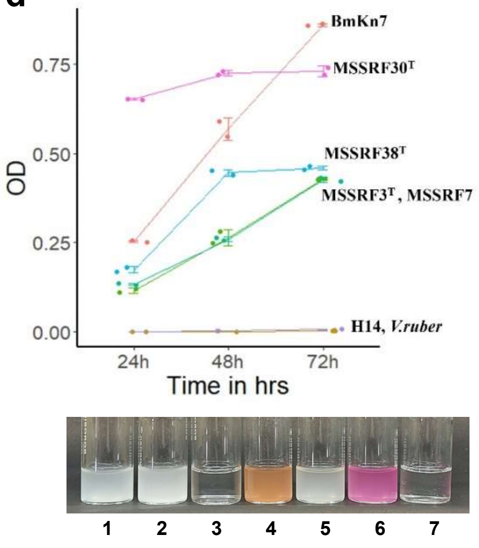

Figure S1.

(a) The standard graph of gluconic acid derived from HPLC and the predicted concentration of gluconic acid produced by MSSRF30^T at 4, 8, and 12 hours post-inoculation (HPI).

(b) The release of inorganic phosphate (Pi) by MSSRF30^T was estimated using the ammonium molybdate-ascorbic acid method. The difference between the inoculated and control was approximately 121 nmol, an average of 3 replicates, 12 HPI.

The bacterial growth in modified M9 minimal medium containing 20% natural seawater, supplemented with NH_4Cl **(c)** or ACC **(d)** as the sole nitrogen source. Labels: 1, MSSRF30^T; 2, Bmkn7 (positive control); 3, *V. parahaemolyticus* H14 (negative control); 4, MSSRF3^T; 5, MSSRF7; 6, MSSRF38^T; 7, MSSRF10.

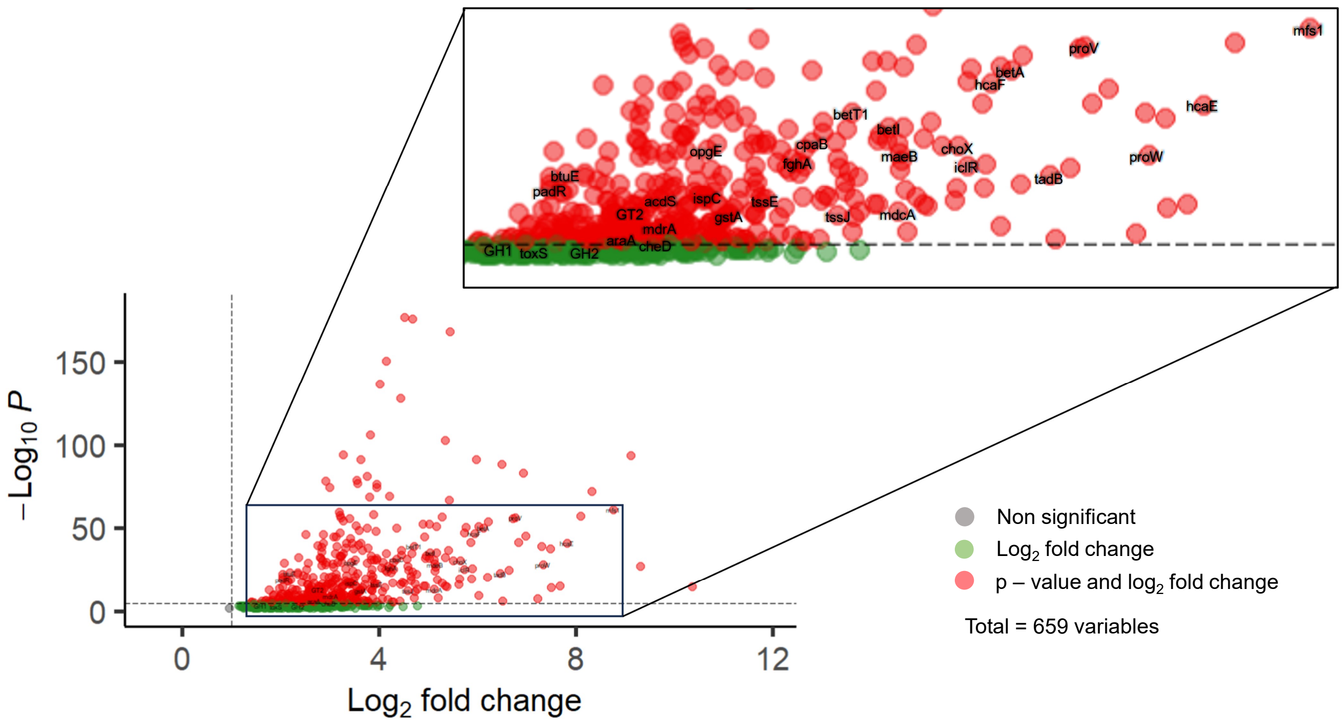

Predicted functions	Upregulated gene
Chemotaxis	*cheD*
Adhesion	*tadB, cpaB, GT2*
Carbohydrate metabolism	*araA*
CAZymes	*GH1, GH2*
Dicarboxylic metabolism	*mdcA, maeB*
Aromatic compound metabolism	*hcaF*
Gene regulation	*toxS*
Quorum sensing	*padR*
Biofilm formation	*ispC, opgE*
Oxidative stress	*btuE, fghA,gstA*
Glycine betaine biosynthesis	*betA, betI, betT1, choX, proV, proW*
PGPR-related functions	*acdS*
T6SS-related genes	*tssJ, tssE*
Multidrug efflux/detoxification	*mdrA*
Transporters	*mfs1*
Other transcriptional regulators	*iclR*

Figure S2. Significantly upregulated genes in MSSRF30^{T} during the early pokkali rice root colonization. The volcano plot shows the distribution of upregulated genes that are statistically significant (p-value < 0.05; corresponding to -log10(0.05) ≈ 1.3). The magnified region highlights selected relevant genes predicted for plant association, as detailed in the table below. An extended list of upregulated genes can be found in supplementary Table S2.

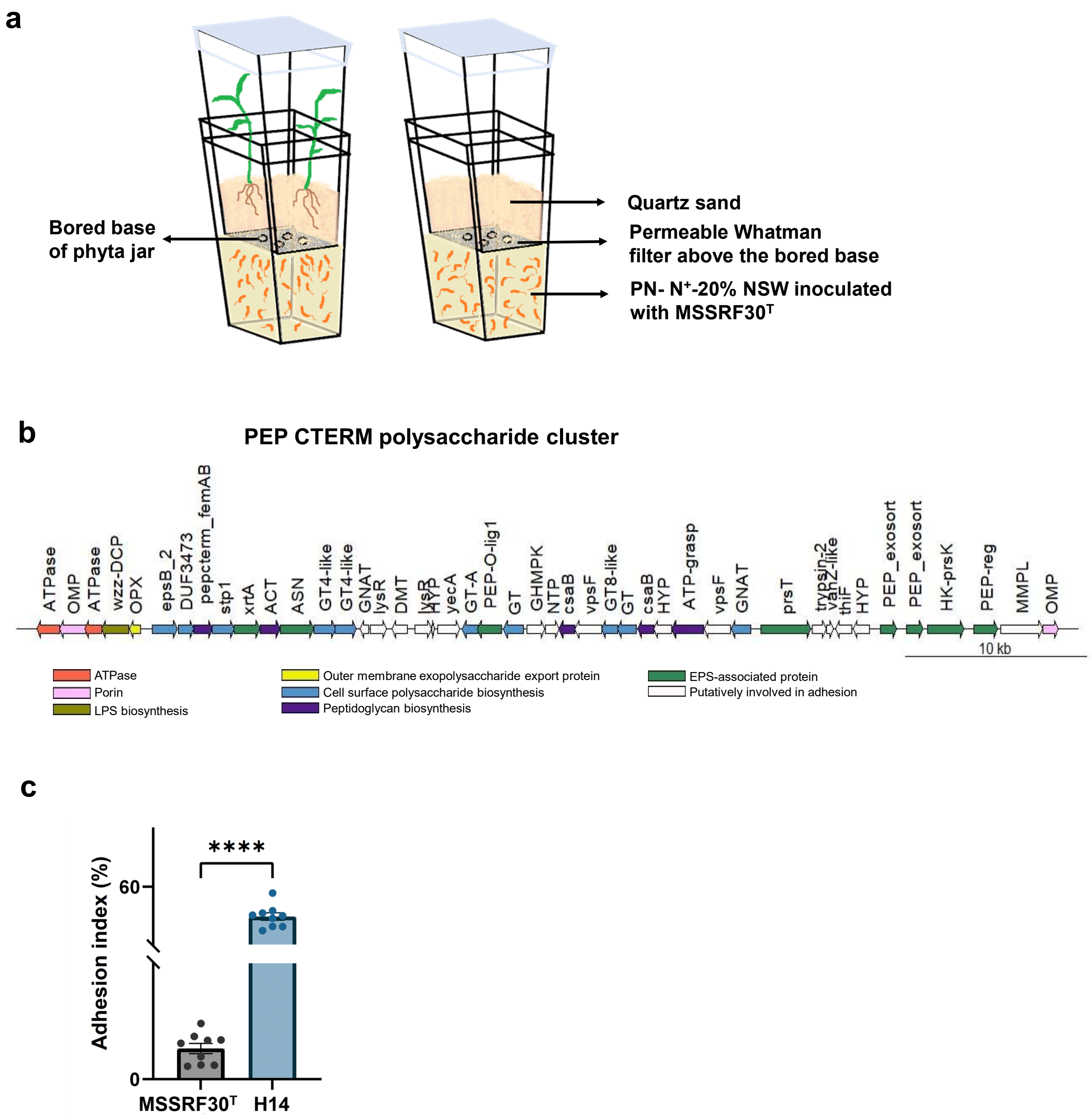

Figure S3.

(a) A graphical representation of the chemotaxis experiment designed to investigate chemotaxis in MSSRF30^{T} under the plant host and non-host conditions. The Whatman filter, as the permeable barrier seen above the bored base of the top phyta jar, separates the plant nutrient solution containing MSSRF30^{T} from the quartz sand layer of the top phyta jar. In the host condition, plants are grown in quartz sand, whereas the non-host condition consists of sand alone. The barrier allows for the diffusion of nutrients and bacterial entry, while preventing direct physical interaction, enabling the assessment of the chemotactic ability of MSSRF30^{T} to cross the barrier and reach the plant, as evidenced by its recovery from the plant roots but not from the non-host control, quartz sand.

(b) The PEP-CTERM gene cluster predicted in MSSRF30^{T}.

(c) The adhesion index of MSSRF30^{T} and *V. parahaemolyticus* H14 to the 8-day-old confluent Caco-2 cells post 2 hours of incubation.

a

Growth in sugars, sugar alcohol, dicarboxylic acids			
Fructose	+++	Trehalose	-
Cellobiose	+++	Raffinose	-
Arabinose	+++	Mannose	-
Xylose	+++	Maltose	-
Galactose	+++	Lactose	-
Sucrose	+++	Mannitol	+++
Glucose	++	Malic acid	++
Rhamnose	+	Succinic acid	++

b

Amino acids			
Proline	+++	Threonine	-
Histidine	+	Asparagine	-
Serine	++	Glutamine	-
Tryptophan	++	Methionine	-
Arginine	++	Isoleucine	-
Lysine	+	Leucine	-
Glycine	-	Phenylalanine	-
Alanine	-	Cysteine	-
Valine	-	Putrescine	-

+/- representative of the OD_{600} values - **+++** : above 0.3 ; **++** : between 0.2 and 0.3 ; **+** : between 0.1 and 0.2 ; **-** : no growth

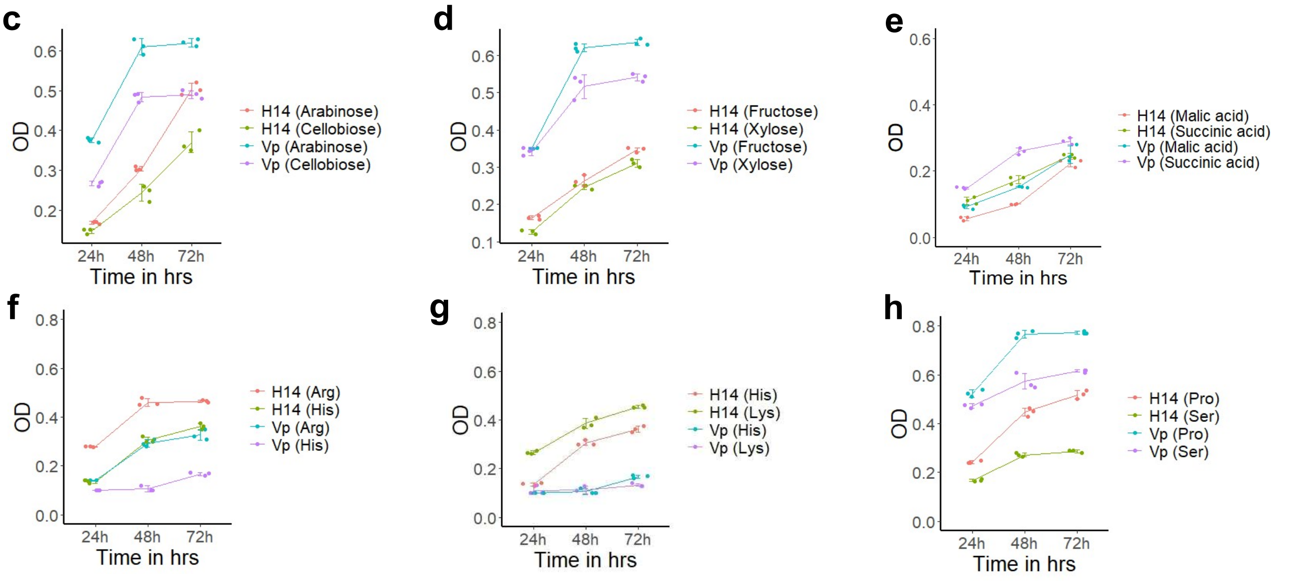

Figure S4: Substrate utilization profile for MSSRF30^T in the given panel of **(a)** sugars and **(b)** amino acids. The growth profile of MSSRF30^T (Vp) and *V. parahaemolyticus* (H14) in modified M9 medium containing the following substrates **(c)**, arabinose and cellobiose; **(d)**, fructose and xylose; **(e)**, malic acid and succinic acid; **(f)** arginine (Arg) and histidine (His); **(g)** histidine (His) and lysine (Lys); and **(h)**, proline (Pro) and serine (Ser).

a

**Complete genetic systems for the utilisation of**	***Vibrio* genomes**								
	**Vp**	**Vc**	**Vpa**	**Vco**	**Vb**	**Vh**	**Vv**	**Va1**	**Af**
Arabinose	■								
Xylose	■				■				
Rhamnose	■								
Myo-inositol	■			■		■			
Gluconate	■			■					
Malonate	■								
Galactarate	■								
Glucarate	■								

■ Presence, □ Absence

b

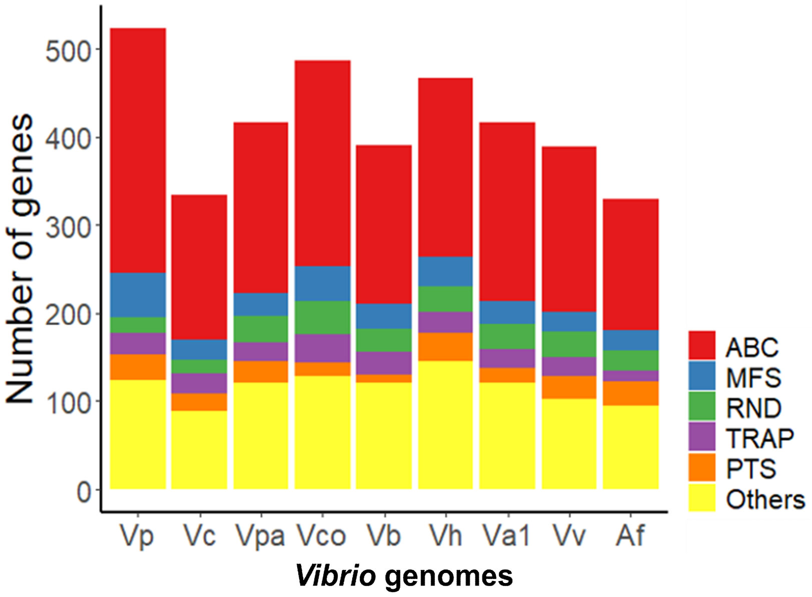

Figure S5.
(a) Complete genetic systems for plant-substrate utilization predicted in the genomes of MSSRF30^{T} and other host-associated *Vibrio* species.
(b) Putative transport proteins predicted in the genomes of MSSRF30^{T} and other host-associated *Vibrio* species.

Abbreviations: Vp, MSSRF30^{T}; **Vc,** *V. cholerae* O1 biovar El Tor str. N16961; **Vpa,** *V. parahaemolyticus* RIMD 2210633; **Vco,** *V. coralliilyticus* Rb102; **Vb,** *V. breoganii* CAIM 1829; **Vh,** *V. harveyi* SB1; **Vv,** *V. vulnificus* ATCC 27562; **Va1,** *V. alginolyticus* NBRC 15630; **Af,** *Aliivibrio fischeri* ES114

a

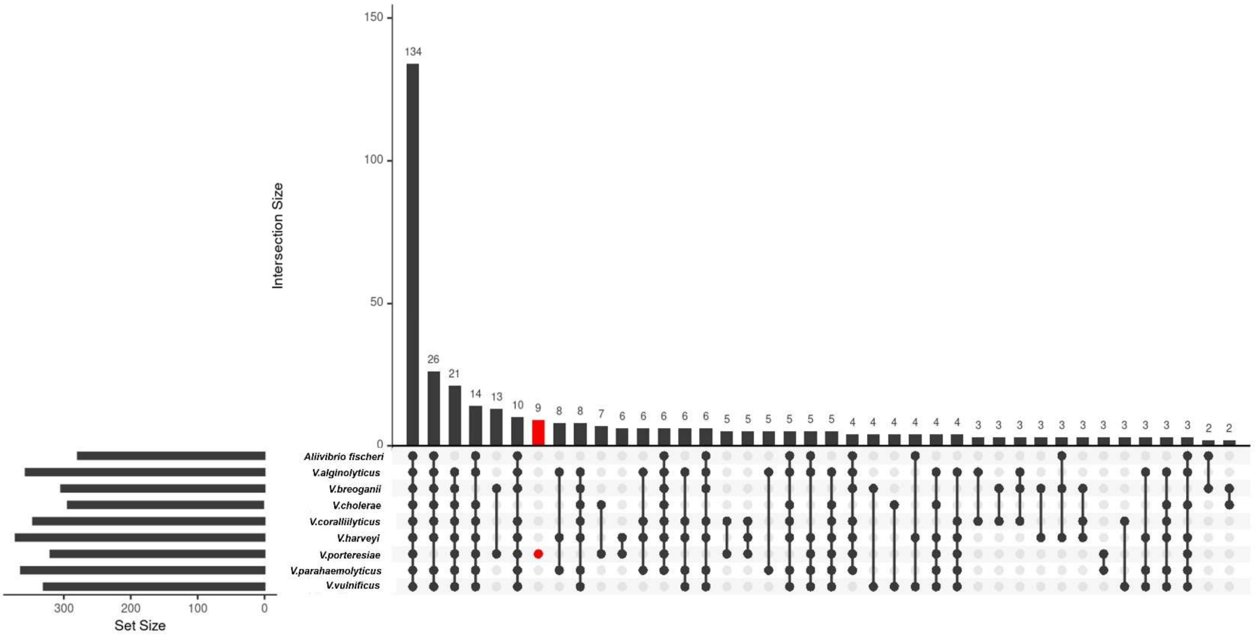

b

S.No	Transport and metabolism of beta-glucosides and glucose	
1	WP_261892410.1	beta-glucoside-specific PTS transporter subunit IIABC
2	WP_261894452.1	beta-glucoside-specific PTS transporter subunit IIABC
3	WP_261895306.1	beta-glucoside-specific PTS transporter subunit IIABC
4	WP_261896625.1	PTS transporter subunit EIIC
5	WP_261896628.1	glucose PTS transporter subunit IIA
6	WP_318757261.1	PTS transporter subunit EIIC
	**Sugar-phosphate transporters**	
7	WP_261897464.1	MFS transporter
8	WP_261896747.1	MFS transporter
	**Glucose transporter**	
9	WP_261897628.1	glucose PTS transporter subunit IIA
	**Sodium-galactoside symporters**	
10	WP_261895534.1	MFS transporter
11	WP_261896033.1	MFS transporter
12	WP_261896447.1	MFS transporter
13	WP_261896668.1	MFS transporter
	**Vitamin B12-binding transporters**	
14	WP_261892600.1	ABC transporter substrate-binding protein
15	WP_261893279.1	ABC transporter substrate-binding protein
16	WP_261895656.1	ABC transporter substrate-binding protein
17	WP_261895885.1	ABC transporter substrate-binding protein
18	WP_261897037.1	ABC transporter substrate-binding protein

	Aromatic compound metabolism	
19	WP_261893297.1	ABC transporter substrate-binding protein
	**Metabolite transporter in nif cluster**	
20	WP_261896716.1	AEC family transporter
	**Murein glycan degradation**	
21	WP_261893306.1	transporter substrate-binding domain-containing protein
22	WP_261894184.1	transporter substrate-binding domain-containing protein
23	WP_318757808.1	transporter substrate-binding domain-containing protein
	**Malonate utilization**	
24	WP_261895522.1	AEC family transporter
25	WP_261895981.1	ABC transporter substrate-binding protein
	**Possible redox transport**	
26	WP_261895326.1	DMT family transporter
27	WP_261897287.1	DMT family transporter
	**Active transport of solutes**	
28	WP_261896833.1	ABC transporter substrate-binding protein

Figure S6.

(a) Upset plot shows MSSRF30^{T} genome harbors unique 28 transporters belonging to 9 orthogroups, marked in red.

(b) Table showing the distinct 28 transporters belonging to 9 orthogroups identified in the Upset plot. Six transporters, including PTS subunits, that are part of carbohydrate-processing enzyme gene clusters (refer to main Figure 6b). Other predicted sugar-phosphate transporters, a glucose PTS transporter, and sodium-galactoside symporters may facilitate a flexible sugar uptake system, aiding in rhizosphere adaptation. An ABC transporter (no. 19), highly upregulated in early root colonization (log2FC = 6.5, refer to supplementary table S2), is encoded within an aromatic compound metabolism cluster. An AEC family transporter (no. 20), identified to be co-localized within the core nif genes, possibly functions in metabolite transport under nitrogen deficiency. Three transporters (nos. 21,22,23) are predicted to be associated with murein glycan degradation. This may function in cell wall remodeling during root colonization or biofilm formation. A transporter (no.24) within the malonate utilization operon, a leucine-binding protein (no.25), and one for solute transport (no.28) suggest the capacity to metabolize root exudate components. Two inner membrane proteins (nos. 26,27) of unknown function may support redox and transport roles under environmental stress. These transporters indicate a metabolic plasticity and environmental adaptability consistent with plant root habitats.

Reference strains	Abbreviation	GH families	PL families
Vibrio porteresiae MSSRF30^T	Vp	26	6
Vibrio cholerae O1 biovar El Tor str. N16961	Vc	18	0
Vibrio parahaemolyticus RIMD 2210633	Vpa	18	2
Vibrio coralliilyticus Rb102	Vco	22	0
Vibrio breoganii CAIM 1829	Vb	23	4
Vibrio harveyi SB1	Vh	22	4
Vibrio alginolyticus NBRC 15630	Va2	19	2
Vibrio vulnificus ATCC 27562	Vv	23	0
Aliivibrio fischeri ES114	Af	19	1

Figure S7. Table showing the number of glycosyl hydrolase (GH) and polysaccharide lyase (PL) families predicted in the genomes of MSSRF30^T and with other pathogenic, squid-symbiotic, and algal-associated vibrios.
