# Supplementary material for: Wild rice-associated Vibrio promotes plant growth and exhibits genomic and phenotypic plasticity for plant adaptations
Source: mSystems. 2025 Oct 27;10(11):e00910-25. doi: 10.1128/msystems.00910-25 (PMC12625758; doi:10.1128/msystems.00910-25)
Supplement: Table S1 — MSSRF30T genome features. [file msystems.00910-25-s0004.pdf]

Supplementary Tables

Table S1

(a) General genome features of MSSRF30^T

Chromosomes	Size
1	3625983
2	1872626
**Feature**	**Value**
Genome size (bp)	5498609
GC content	44.8
No. of CDS	4793
Assigned functions	4350
Hypothetical proteins	443
Prophage functions	4
Integrons	3
Integrases	6
IS elements	44
**RNA elements**	
rRNA	34
tRNA	119

(b) Functional gene categories based on Clusters of Orthologous Groups of Proteins (COG) database

Category	Code	Description	*Vibrio porteresiae* MSSRF30^T	*Aliivibrio fischeri* ES114	*Vibrio cholerae* O1 biovar El Tor str. N16961	*Vibrio breoganii* LMG 23858T
Metabolism	C	Energy production and conversion	272	200	209	212
	**E**	**Amino acid transport and metabolism**	**256**	**173**	**195**	**210**
	F	Nucleotide transport and metabolism	140	120	133	122

	**G**	**Carbohydrate transport and metabolism**	**218**	**136**	**150**	**151**
	H	Coenzyme transport and metabolism	194	155	181	155
	I	Lipid transport and metabolism	88	62	79	73
	**P**	**Inorganic ion transport and metabolism**	**282**	**202**	**239**	**232**
	Q	Secondary metabolites biosynthesis, transport and catabolism	50	23	45	39
Cellular processes and signaling	D	Cell cycle, cell division, chromosome partitioning	55	51	51	45
	M	Cell wall/membrane/envelope biogenesis	226	243	210	224
	N	Cell motility	50	57	53	1
	O	Posttranslational modification, protein turnover, chaperones	104	104	110	98
	T	Signal transduction mechanisms	179	148	191	74
	U	Intracellular trafficking, secretion, and vesicular transport	97	107	78	76
	V	Defense mechanisms	50	56	58	63
DNA, RNA AND protein synthesis	A	RNA processing and modification	1	1	1	2
	J	Translation, ribosomal structure and biogenesis	217	199	208	203

	K	Transcription	383	257	254	262
	L	Replication, recombination and repair	166	129	168	157
Poorly characterized	HYP	Hypothetical	175	219	195	198
	S	Function unknown	839	710	709	698

Plant-associated genes predicted in the MSSRF30^{T} genome

(c) Nitrogen fixation

Vp_protein_ID	Chr no.	Orientation	Annotation	Symbol
**Genes putatively involved in nitrogen fixation**				
WP_261893887.1	1	minus	serine O-acetyltransferase	*cysE*
WP_261892354.1	1	plus	CCE_0567 family metalloprotein	--
WP_261892991.1	1	minus	ATP-dependent protease ATP-binding subunit ClpX	*clpX*
WP_261893244.1	1	minus	RnfH family protein	*rnf*
WP_261896013.1	1	minus	ferredoxin--NADP reductase	--
WP_261893433.1	1	minus	Fe-S cluster assembly protein IscX	*iscX*
WP_261893435.1	1	minus	ISC system 2Fe-2S type ferredoxin	*fdx*
WP_261893437.1	1	minus	Fe-S protein assembly chaperone HscA	*hscA*
WP_261893438.1	1	minus	co-chaperone HscB	*hscB*
WP_261893440.1	1	minus	iron-sulfur cluster assembly protein IscA	*iscA*
WP_261893442.1	1	minus	Fe-S cluster assembly scaffold IscU	*iscU*
WP_261893443.1	1	minus	IscS subfamily cysteine desulfurase	--
WP_261893444.1	1	minus	Fe-S cluster assembly transcriptional regulator IscR	*iscR*
WP_261893479.1	1	plus	YfhL family 4Fe-4S dicluster ferredoxin	--
WP_261893480.1	1	minus	oxygen-insensitive NADPH nitroreductase	*nfsA*
**Cluster 1 – Electron transport and regulators**				
WP_261896644.1	2	minus	flavodoxin	*nifF*
WP_261896646.1	2	minus	RnfH family protein	*rnfH*
WP_261896647.1	2	minus	electron transport complex subunit E	*rnfE*
WP_261896648.1	2	minus	RnfABCDGE type electron transport complex subunit G	*rnfG*
WP_261896649.1	2	minus	RnfABCDGE type electron transport complex subunit D	*rnfD*
WP_261896650.1	2	minus	electron transport complex subunit RsxC	*rsxC*
WP_261896651.1	2	minus	RnfABCDGE type electron transport complex subunit B	*rnfB*
WP_261896652.1	2	minus	electron transport complex subunit RsxA	*rsxA*
WP_261896653.1	2	plus	nitrogen fixation negative regulator NifL	*nifL*
WP_261896654.1	2	plus	nif-specific transcriptional activator NifA	*nifA*
WP_261896655.1	2	plus	nitrogenase cofactor biosynthesis protein NifB	*nifB*
WP_261896656.1	2	plus	4Fe-4S dicluster domain-containing protein	--
WP_261896657.1	2	plus	nitrogen fixation protein NifQ	*nifQ*
WP_261896658.1	2	minus	YagU family protein	--
**Cluster 2 – Nitrogenase structure, function, maturation, and stabilization**				
WP_261896700.1	2	minus	peptidylprolyl isomerase	*nifM*
WP_261896701.1	2	minus	nitrogen fixation protein NifZ	*nifZ*
WP_261896702.1	2	minus	nitrogenase-stabilizing/protective protein NifW	*nifW*
WP_261896703.1	2	minus	homocitrate synthase	*nifV*
WP_261896704.1	2	minus	cysteine desulfurase NifS	*nifS*
WP_261896705.1	2	minus	Fe-S cluster assembly protein NifU	*nifU*
WP_261896706.1	2	minus	ferredoxin III, nif-specific	*fdxB*
WP_261896707.1	2	minus	NifB/NifX family molybdenum-iron cluster-binding protein	*nifX*
WP_261896708.1	2	minus	nitrogenase iron-molybdenum cofactor biosynthesis protein NifN	*nifN*
WP_261896709.1	2	minus	nitrogenase iron-molybdenum cofactor biosynthesis protein NifE	*nifE*
WP_261896710.1	2	minus	hypothetical protein	--
WP_261896711.1	2	minus	NifB/NifX family molybdenum-iron cluster-binding protein	*nifY*
WP_261896712.1	2	minus	putative nitrogen fixation protein NifT	*nifT*
WP_261896713.1	2	minus	nitrogenase molybdenum-iron protein subunit beta	*nifK*
WP_261896714.1	2	minus	nitrogenase molybdenum-iron protein alpha chain	*nifD*
WP_261896715.1	2	minus	nitrogenase iron protein	*nifH*
WP_261897614.1	2	minus	NirD/YgiW/YdeI family stress tolerance protein	*nirD*
**Cluster 3 – Molybdate transporter genes**				
WP_261897615.1	2	plus	molybdate ABC transporter substrate-binding protein	*modA*
WP_261897961.1	2	plus	molybdate ABC transporter permease subunit	*modB*

WP_261897616.1	2	plus	molybdenum ABC transporter ATP-binding protein ModC	*modC*

(d) ACC deaminase

Vp_protein_ID	Chr no.	Orientation	Annotation	Symbol
WP_261892913.1	1	minus	1-aminocyclopropane-1-carboxylate deaminase	*acdS*
WP_261892914.1	1	plus	Lrp/AsnC ligand binding domain-containing protein	*lrp*

(e) Phosphate solubilization and uptake

Vp_protein_ID	Chr no.	Orientation	Annotation	Symbol
WP_261892133.1	1	plus	alkaline phosphatase D family protein	*phoD*
WP_261892626.1	1	plus	HAD family acid phosphatase	*aphA2*
WP_261893010.1	1	minus	citrate:proton symporter	
WP_261893468.1	1	minus	phosphate signaling complex protein PhoU	*phoU*
WP_261893469.1	1	minus	phosphate ABC transporter ATP-binding protein PstB	*pstB*
WP_261893470.1	1	minus	phosphate ABC transporter permease PstA	*pstA*
WP_261893471.1	1	minus	ABC transporter permease subunit	--
WP_261893472.1	1	plus	polyphosphate kinase 1	*ppk1*
WP_261893473.1	1	plus	exopolyphosphatase	*ppx*
WP_261893474.1	1	minus	phosphate ABC transporter substrate-binding protein PstS family protein	--
WP_261893475.1	1	minus	phosphate regulon sensor histidine kinase PhoR	*phoR*
WP_261893476.1	1	minus	phosphate regulon transcriptional regulator PhoB	*phoB*
WP_261895325.1	1	minus	acid phosphatase AphA	*aphA1*
WP_261895381.1	1	plus	GMC family oxidoreductase	*gdh*
WP_261895382.1	1	plus	cytochrome c	*cytC*
WP_261895384.1	1	plus	gluconate 2-dehydrogenase subunit 3 family protein	GA2DH-γ
WP_261895385.1	1	plus	GMC family oxidoreductase	GA2DH-f
WP_261895387.1	1	plus	cytochrome c	*GA2DH-cytC*
WP_261896233.1	1	minus	phosphate ABC transporter ATP-binding protein PstB	*pstB1*
WP_261895605.1	1	minus	phosphate ABC transporter permease PstA	*pstA1*
WP_261895607.1	1	minus	phosphate ABC transporter permease subunit PstC	*pstC1*
WP_261895608.1	1	minus	phosphate ABC transporter substrate-binding protein PstS family protein	*pstS*
WP_261896678.1	2	minus	alkaline phosphatase	*alkPPc*
WP_261897084.1	2	minus	phosphate ABC transporter ATP-binding protein PstB	*pstB3*
WP_261897996.1	2	minus	phosphate ABC transporter permease PstA	*pstA3*
WP_261897085.1	2	minus	phosphate ABC transporter permease subunit PstC	*pstC2*
WP_261897235.1	2	minus	alkaline phosphatase family protein	*alkP*
WP_261897398.1	2	minus	cytochrome c	GA2DH-*cytC*
WP_261897399.1	2	minus	GMC family oxidoreductase	GA2DH-f
WP_261897400.1	2	minus	gluconate 2-dehydrogenase subunit 3 family protein	GA2DHγ
WP_261897953.1	2	minus	glucose-6-phosphate dehydrogenase	*gdh2*

Secretion systems

(f) T3SS

Vp_protein_ID	Chr no.	Orientation	Annotation	Symbol
WP_261892995.1	1	plus	sigma-54 dependent transcriptional regulator	*hrpR*
WP_261893328.1	1	plus	pectate lyase	*hrpW*
WP_261893330.1	1	plus	AvrE-family type 3 secretion system effector	*avrE1*
WP_261893332.1	1	plus	transposase	Tn-IS200
WP_261893333.1	1	minus	HrpE/YscL family type III secretion apparatus protein	*hrpE*
WP_261893334.1	1	minus	hypothetical protein	T3SP/HYP
WP_261893335.1	1	minus	type III secretion inner membrane ring lipoprotein SctJ	*sctJ*
WP_261893336.1	1	minus	type III secretion system inner rod subunit SctI	*sctI*
WP_261893337.1	1	minus	harpin HrpZ family protein	*hrpZ*
WP_261893339.1	1	minus	hypothetical protein	*hrpA*
WP_261893341.1	1	plus	hypothetical protein	--
WP_261893343.1	1	plus	PAS domain-containing protein	PAS
WP_261893344.1	1	plus	response regulator transcription factor	--
WP_261893345.1	1	plus	hypothetical protein	*pelA*
WP_261893346.1	1	plus	hypothetical protein	*chap*
WP_261893347.1	1	plus	type III secretion system gatekeeper subunit SctW	*sctW*
WP_261893348.1	1	plus	type III secretion system export apparatus subunit SctV	*sctV*
WP_261893350.1	1	plus	GGDEF domain-containing protein	GGDEF
WP_261893352.1	1	plus	FHA domain-containing protein	*sctD*
WP_261893354.1	1	plus	FliI/YscN family ATPase	*yscN*
WP_261893356.1	1	plus	hypothetical protein	*yscO-like*
WP_261893357.1	1	plus	type III secretion system HrpP C-terminal domain-containing protein	*hrpP*
WP_261893358.1	1	plus	FliM/FliN family flagellar motor switch protein	*fliM/fliN*
WP_261893360.1	1	plus	type III secretion system export apparatus subunit SctR	*sctR*
WP_261893362.1	1	plus	type III secretion system export apparatus subunit SctS	*sctS*
WP_261893364.1	1	plus	type III secretion system export apparatus subunit SctT	*sctT*
WP_261893366.1	1	plus	type III secretion system export apparatus subunit SctU	*sctU*
WP_261893368.1	1	plus	RNA polymerase sigma factor	*hrpL*
WP_261893370.1	1	plus	type III secretion system chaperone	T3SC
WP_261893372.1	1	plus	type III secretion protein HrpF	*hrpF*
WP_261893374.1	1	plus	hypothetical protein	--
WP_261893376.1	1	plus	type III secretion system outer membrane ring subunit SctC	*sctC*
WP_261893377.1	1	plus	hypothetical protein	*hrpT*
WP_261893379.1	1	plus	hypothetical protein	--
WP_261897383.1	2	plus	type III effector HrpK domain-containing protein	*hrpK*
WP_261896515.1	2	minus	HopJ type III effector protein	*hopJ*

(g) T6SS

Vp_protein_ID	Chr no.	Orientation	Annotation	Symbol
**Auxiliary cluster 1**				
WP_261892120.1	1	minus	hypothetical protein	--
WP_261892121.1	1	minus	hypothetical protein	*lysM* DCP
WP_261892122.1	1	minus	DUF4123 domain-containing protein	DUF4123
WP_261892124.1	1	minus	type VI secretion system tip protein TssI/VgrG	*vgrG-1*
WP_261892126.1	1	minus	Hcp family type VI secretion system effector	*hcp*

**Auxiliary cluster 2**				
WP_261897126.1	2	minus	hypothetical protein	DUF6708
WP_261897127.1	2	minus	hypothetical protein	--
WP_261897128.1	2	minus	LysM domain-containing protein	*lysM* DCP
WP_261897129.1	2	minus	DUF4123 domain-containing protein	DUF4123
WP_261897130.1	2	minus	type VI secretion system tip protein VgrG	*vgrG*-2
WP_261897131.1	2	minus	Hcp family type VI secretion system effector	*hcp*
**Large gene cluster**				
WP_261897247.1	2	plus	sel1 repeat family protein	*sel1*-family
WP_261897248.1	2	plus	type VI secretion system contractile sheath small subunit	*tssB*
WP_261897249.1	2	plus	type VI secretion system contractile sheath large subunit	*tssC*
WP_261897250.1	2	plus	type VI secretion system baseplate subunit TssE	*tssE*
WP_261897251.1	2	plus	type VI secretion system baseplate subunit TssF	*tssF*
WP_261897252.1	2	plus	type VI secretion system baseplate subunit TssG	*tssG*
WP_261897253.1	2	plus	type VI secretion system-associated FHA domain protein TagH	*tagH*
WP_261897944.1	2	plus	type VI secretion system lipoprotein TssJ	*tssJ*
WP_261897254.1	2	plus	type VI secretion system baseplate subunit TssK	*tssK*
WP_261897255.1	2	plus	type IVB secretion system protein IcmH/DotU	*icmH*
WP_261897256.1	2	plus	type VI secretion system ATPase TssH	*tssH*
WP_261897257.1	2	plus	sigma 54-interacting transcriptional regulator	σ^{54} TR
WP_261897258.1	2	plus	type VI secretion system-associated protein VasI	*vasI*
WP_261897259.1	2	plus	type VI secretion system protein TssA	*tssA*
WP_261897945.1	2	plus	type VI secretion system membrane subunit TssM	*tssM*
WP_261897260.1	2	plus	type VI secretion system ImpA family N-terminal domain-containing protein	*impA*
WP_261897261.1	2	plus	type VI secretion system tip protein VgrG	*vgrG*-3
WP_261897262.1	2	plus	DUF4123 domain-containing protein	DUF4123
WP_261897263.1	2	plus	RHS repeat-associated core domain-containing protein	RHS RCP
WP_261897264.1	2	plus	hypothetical protein	--

Other secretion systems

(h) Type I, II secretion systems, SEC and twin-arginine translocation

Vp_protein_ID	Chr no.	Orientation	Annotation	Symbol
**Type I secretion system**				
WP_261897651.1	2	plus	TolC family protein	*tolC*
WP_261897652.1	2	plus	efflux RND transporter periplasmic adaptor subunit	MFP
WP_261897654.1	2	plus	HlyD family efflux transporter periplasmic adaptor subunit	*hlyD*
**Type II secretion system**				
WP_261893977.1	1	minus	type II secretion system protein N	*gspN*
WP_261893979.1	1	minus	type II secretion system protein M	*gspM*
WP_261893981.1	1	minus	type II secretion system protein GspL	*gspL*
WP_261893982.1	1	minus	type II secretion system minor pseudopilin GspK	*gspK*
WP_261893983.1	1	minus	type II secretion system minor pseudopilin GspJ	*gspJ*
WP_261893984.1	1	minus	type II secretion system minor pseudopilin GspI	*gspI*
WP_261893985.1	1	minus	type II secretion system minor pseudopilin GspH	*gspH*

WP_261893986.1	1	minus	type II secretion system major pseudopilin GspG	*gspG*
WP_261893987.1	1	minus	type II secretion system inner membrane protein GspF	*gspF*
WP_261893988.1	1	minus	type II secretion system ATPase GspE	*gspE*
WP_261893989.1	1	minus	type II secretion system secretin GspD	*gspD*
WP_261893990.1	1	minus	type II secretion system protein GspC	*gspC*
**SEC and TAT systems**				
WP_261894069.1	1	plus	TatD family hydrolase	--
WP_261894070.1	1	minus	twin-arginine translocase subunit TatC	*tatC*
WP_261894071.1	1	minus	Sec-independent protein translocase protein TatB	*tatB*
WP_261894072.1	1	minus	Sec-independent protein translocase subunit TatA	*tatA*
WP_261894946.1	1	plus	TatD family hydrolase	--
WP_261895361.1	1	plus	YchF/TatD family DNA exonuclease	--
WP_261893181.1	1	minus	SecY-interacting protein	*syd*
WP_261893452.1	1	minus	protein translocase subunit SecF	*secF*
WP_261893454.1	1	minus	protein translocase subunit SecD	*secD*
WP_068714755.1	1	minus	preprotein translocase subunit YajC	*yajC*
WP_261893457.1	1	minus	tRNA guanosine(34) transglycosylase Tgt	*tgt*
WP_261893458.1	1	minus	tRNA preQ1(34) S-adenosylmethionine ribosyltransferase-isomerase QueA	*queA*

(i) Quorum sensing systems

Vp_protein_ID	Chr no.	Orientation	Annotation	Symbol
**LuxS-AI-2 system**				
WP_261894852.1	1	plus	S-ribosylhomocysteine lyase	*luxS*
WP_261897611.1	2	plus	autoinducer 2-binding periplasmic protein LuxP	*luxP*
WP_261897612.1	2	plus	quorum-sensing autoinducer 2 sensor kinase/phosphatase LuxQ	*luxQ*
WP_261893080.1	1	minus	quorum-sensing phosphorelay protein LuxU	*luxU*
WP_261893081.1	1	minus	quorum-sensing sigma-54 dependent transcriptional regulator LuxO	*luxO*

(j) Motility

Vp_protein_ID	Chr no.	Orientation	Annotation	Symbol
WP_261896324.1	1	plus	flagellar basal body-associated protein FliL	*fliL*
WP_261895106.1	1	minus	flagellar assembly protein FlgT	*flgT*
WP_261895107.1	1	plus	FlgO family outer membrane protein	*flgO*
WP_261895108.1	1	plus	flagellar assembly lipoprotein FlgP	*flgP*
WP_261895109.1	1	minus	flagellar export chaperone FlgN	*flgN*
WP_261895110.1	1	minus	flagellar biosynthesis anti-sigma factor FlgM	*flgM*
WP_261895111.1	1	minus	flagellar basal body P-ring formation chaperone FlgA	*flgA*
WP_261895114.1	1	plus	flagellar basal body rod protein FlgB	*flgB*
WP_261895115.1	1	plus	flagellar basal body rod protein FlgC	*flgC*
WP_261895117.1	1	plus	flagellar hook assembly protein FlgD	*flgD*
WP_261895119.1	1	plus	flagellar hook protein FlgE	*flgE*
WP_261895121.1	1	plus	flagellar basal-body rod protein FlgF	*flgF*
WP_261895123.1	1	plus	flagellar basal-body rod protein FlgG	*flgG*
WP_261895124.1	1	plus	flagellar basal body L-ring protein FlgH	*flgH*
WP_261895126.1	1	plus	flagellar basal body P-ring protein FlgI	*flgI*
WP_261895128.1	1	plus	flagellar assembly peptidoglycan hydrolase FlgJ	*flgJ*
WP_261895130.1	1	plus	flagellar hook-associated protein FlgK	*flgK*
WP_261895131.1	1	plus	flagellar hook-associated protein FlgL	*flgL*

WP_261895132.1	1	plus	flagellin	--
WP_261895134.1	1	plus	flagellin	--
WP_261895167.1	1	minus	flagellin	--
WP_261895168.1	1	plus	flagellin	--
WP_261895170.1	1	plus	flagellin	--
WP_261895172.1	1	plus	flagellin	--
WP_261895173.1	1	plus	flagellar protein FlaG	*flaG*
WP_261895174.1	1	plus	flagellar filament capping protein FliD	*fliD*
WP_261895175.1	1	plus	flagellar protein FliT	*fliT*
WP_261895177.1	1	plus	flagellar export chaperone FliS	*fliS*
WP_261895178.1	1	plus	sigma-54 dependent transcriptional regulator	--
WP_261895179.1	1	plus	ATP-binding protein	--
WP_261895180.1	1	plus	sigma-54 dependent transcriptional regulator	--
WP_261896222.1	1	plus	flagellar hook-basal body complex protein FliE	*fliE*
WP_261895182.1	1	plus	flagellar basal-body MS-ring/collar protein FliF	*fliF*
WP_261895183.1	1	plus	flagellar motor switch protein FliG	*fliG*
WP_261895184.1	1	plus	flagellar assembly protein FliH	*fliH*
WP_261895186.1	1	plus	flagellar protein export ATPase FliI	*fliI*
WP_261895187.1	1	plus	flagellar export protein FliJ	*fliJ*
WP_261895188.1	1	plus	flagellar hook-length control protein FliK	*fliK*
WP_261895189.1	1	plus	flagellar basal body-associated protein FliL	*fliL*
WP_261895190.1	1	plus	flagellar motor switch protein FliM	*fliM*
WP_261895191.1	1	plus	flagellar motor switch protein FliN	*fliN*
WP_261896223.1	1	plus	flagellar biosynthetic protein FliO	*fliO*
WP_261895192.1	1	plus	flagellar type III secretion system pore protein FliP	*fliP*
WP_068712599.1	1	plus	flagellar biosynthesis protein FliQ	*fliQ*
WP_261895193.1	1	plus	flagellar biosynthetic protein FliR	*fliR*
WP_261895195.1	1	plus	flagellar biosynthesis protein FlhB	*flhB*
WP_261895249.1	1	plus	flagellar biosynthesis protein FlhA	*flhA*
WP_261895251.1	1	plus	flagellar biosynthesis protein FlhF	*flhF*
WP_261895874.1	1	plus	flagellin	--
WP_261893193.1	1	minus	flagellar motor protein MotB	*motB*
WP_261893194.1	1	minus	flagellar motor protein PomA	*pomA*
WP_261897102.1	2	plus	flagellar brake protein	--

Chemotaxis

WP_261894295.1	1	plus	methyl-accepting chemotaxis protein	--
WP_261894473.1	1	minus	methyl-accepting chemotaxis protein	--
WP_261894623.1	1	minus	chemotaxis protein CheX	*cheX*
WP_261895112.1	1	plus	chemotaxis protein CheV	*cheV*
WP_261895156.1	1	plus	methyl-accepting chemotaxis protein	--
WP_000697869.1	1	plus	chemotaxis response regulator CheY	*cheY*
WP_261895257.1	1	plus	chemotaxis protein CheA	*cheA*
WP_261895258.1	1	plus	chemotaxis response regulator protein-glutamate methylesterase	--
WP_261895261.1	1	plus	chemotaxis protein CheW	*cheW*
WP_261895263.1	1	plus	chemotaxis protein CheW	*cheW*
WP_261895332.1	1	plus	methyl-accepting chemotaxis protein	--
WP_261895372.1	1	plus	chemotaxis protein CheV	*cheV*
WP_261895421.1	1	plus	methyl-accepting chemotaxis protein	--
WP_261895422.1	1	plus	methyl-accepting chemotaxis protein	--
WP_261895454.1	1	plus	chemotaxis protein CheA	*cheA*
WP_261895455.1	1	plus	methyl-accepting chemotaxis protein	--

WP_261895457.1	1	plus	chemotaxis protein CheW	--
WP_261895459.1	1	plus	chemotaxis response regulator protein-glutamate methylesterase	--
WP_261895461.1	1	plus	chemotaxis protein CheD	*cheD*
WP_261895467.1	1	plus	methyl-accepting chemotaxis protein	--
WP_261895485.1	1	plus	methyl-accepting chemotaxis protein	--
WP_315972745.1	1	plus	PAS domain-containing methyl-accepting chemotaxis protein	--
WP_261895911.1	1	minus	methyl-accepting chemotaxis protein	--
WP_261895936.1	1	plus	methyl-accepting chemotaxis protein	--
WP_261895938.1	1	plus	methyl-accepting chemotaxis protein	--
WP_261895945.1	1	plus	methyl-accepting chemotaxis protein	--
WP_261895980.1	1	minus	methyl-accepting chemotaxis protein	--
WP_261896101.1	1	plus	PAS domain-containing methyl-accepting chemotaxis protein	--
WP_261892201.1	1	plus	methyl-accepting chemotaxis protein	--
WP_261892203.1	1	plus	chemotaxis protein CheW	*cheW*
WP_261892205.1	1	plus	chemotaxis protein CheB	*cheB*
WP_261896261.1	1	plus	chemotaxis protein CheA	*cheA*
WP_315972727.1	1	plus	methyl-accepting chemotaxis protein	--
WP_261892326.1	1	plus	methyl-accepting chemotaxis protein	--
WP_261892334.1	1	plus	methyl-accepting chemotaxis protein	--
WP_315972729.1	1	minus	PAS domain-containing methyl-accepting chemotaxis protein	--
WP_261892523.1	1	minus	chemotaxis protein	--
WP_261892649.1	1	plus	methyl-accepting chemotaxis protein	--
WP_315972732.1	1	minus	PAS domain-containing methyl-accepting chemotaxis protein	--
WP_261892884.1	1	plus	methyl-accepting chemotaxis protein	--
WP_261892905.1	1	minus	methyl-accepting chemotaxis protein	--
WP_261892908.1	1	plus	methyl-accepting chemotaxis protein	--
WP_261892910.1	1	plus	methyl-accepting chemotaxis protein	--
WP_261892945.1	1	minus	methyl-accepting chemotaxis protein	--
WP_261893048.1	1	plus	methyl-accepting chemotaxis protein	--
WP_261893251.1	1	plus	methyl-accepting chemotaxis protein	--
WP_261893282.1	1	plus	methyl-accepting chemotaxis protein	--
WP_315972735.1	1	minus	methyl-accepting chemotaxis protein	--
WP_261893652.1	1	minus	methyl-accepting chemotaxis protein	--
WP_261896630.1	2	plus	methyl-accepting chemotaxis protein	--
WP_261896742.1	2	plus	methyl-accepting chemotaxis protein	--
WP_315972752.1	2	minus	PAS domain-containing methyl-accepting chemotaxis protein	--
WP_261896834.1	2	minus	methyl-accepting chemotaxis protein	--
WP_318757885.1	2	plus	type IV pili methyl-accepting chemotaxis transducer N-terminal domain-containing protein	--
WP_261896917.1	2	plus	methyl-accepting chemotaxis protein	--
WP_261896933.1	2	minus	methyl-accepting chemotaxis protein	--
WP_261896953.1	2	plus	methyl-accepting chemotaxis protein	--
WP_261897009.1	2	minus	methyl-accepting chemotaxis protein	--
WP_261897087.1	2	plus	methyl-accepting chemotaxis protein	--
WP_261897118.1	2	minus	methyl-accepting chemotaxis protein	--
WP_261897162.1	2	plus	methyl-accepting chemotaxis protein	--
WP_315972759.1	2	minus	methyl-accepting chemotaxis protein	--
WP_261897299.1	2	plus	PAS domain-containing methyl-accepting chemotaxis protein	--
WP_261897328.1	2	minus	chemotaxis protein	--
WP_318757916.1	2	plus	methyl-accepting chemotaxis protein	--

WP_261897395.1	2	minus	methyl-accepting chemotaxis protein	--
WP_261897411.1	2	plus	methyl-accepting chemotaxis protein	--
WP_261897417.1	2	plus	methyl-accepting chemotaxis protein	--
WP_261897452.1	2	plus	methyl-accepting chemotaxis protein	--
WP_261897469.1	2	minus	methyl-accepting chemotaxis protein	--
WP_261897474.1	2	minus	methyl-accepting chemotaxis protein	--
WP_261897643.1	2	plus	PAS domain-containing methyl-accepting chemotaxis protein	--
WP_261897808.1	2	minus	methyl-accepting chemotaxis protein	--
WP_261897810.1	2	minus	methyl-accepting chemotaxis protein	--
WP_261896380.1	2	plus	methyl-accepting chemotaxis protein	--
WP_261896478.1	2	minus	chemotaxis protein CheC	*cheC*
WP_261896498.1	2	minus	methyl-accepting chemotaxis protein	--
WP_261896503.1	2	minus	methyl-accepting chemotaxis protein	--
WP_261896511.1	2	minus	methyl-accepting chemotaxis protein	--
WP_261896516.1	2	plus	methyl-accepting chemotaxis protein	--

(k) Adhesion

Tad genes

Vp_protein_ID	Chr no.	Orientation	Annotation	Symbol
WP_261896856.1	2	minus	OmpA family protein	*ompA*
WP_261896857.1	2	minus	pilus assembly protein	*tadG*
WP_261896858.1	2	minus	tight adherence pilus pseudopilin TadF	*tadF*
WP_261896859.1	2	minus	pilus assembly protein	*tadE*
WP_261896860.1	2	minus	tetratricopeptide repeat protein	*tadD*
WP_261896861.1	2	minus	type II secretion system F family protein	*tadC*
WP_261896862.1	2	minus	type II secretion system F family protein	*tadB*
WP_261896863.1	2	minus	CpaF family protein	*tadA*
WP_261896864.1	2	minus	type II secretion system protein Z	*tadZ*
WP_261896865.1	2	minus	hypothetical protein	*lspA*
WP_261896866.1	2	minus	pilus assembly protein N-terminal domain-containing protein	*rcpA*
WP_261896867.1	2	minus	Flp pilus assembly protein CpaB	*cpaB*
WP_261896868.1	2	minus	hypothetical protein	*cpaA*
WP_261897988.1	2	minus	Flp family type IVb pilin	*flp*
WP_261896869.1	2	minus	hypothetical protein	*lspA*
WP_261896870.1	2	minus	OmpA family protein	*ompA*
WP_261896871.1	2	minus	hypothetical protein	*tadG*
WP_261896872.1	2	minus	tight adherence pilus pseudopilin TadF	*tadF*
WP_261896873.1	2	minus	pilus assembly protein	*tadE*
WP_261896874.1	2	minus	hypothetical protein	*tadD*
WP_261896875.1	2	minus	type II secretion system F family protein	*tadC*
WP_261897989.1	2	minus	type II secretion system F family protein	*tadB*
WP_261896876.1	2	minus	CpaF family protein	*tadA*
WP_261896877.1	2	minus	hypothetical protein	*atpase*
WP_261896878.1	2	minus	hypothetical protein	*lspA*
WP_261896879.1	2	minus	pilus assembly protein N-terminal domain-containing protein	*rcpA*
WP_261896880.1	2	minus	Flp pilus assembly protein CpaB	*cpaB*
WP_261896881.1	2	minus	prepilin peptidase	*a24*
WP_261896882.1	2	minus	Flp family type IVb pilin	*flp1*
WP_261896883.1	2	minus	Flp family type IVb pilin	*flp2*
WP_261896884.1	2	minus	Flp family type IVb pilin	*flp3*
WP_068718075.1	2	plus	Flp family type IVb pilin	--

WP_261897659.1	2	plus	prepilin peptidase	--
WP_261897660.1	2	plus	Flp pilus assembly protein CpaB	*cpaB*
WP_261897661.1	2	plus	type II and III secretion system protein family protein	--
WP_261897662.1	2	plus	CpaD family pilus assembly protein	--
WP_261897663.1	2	plus	hypothetical protein	--
WP_261897664.1	2	plus	CpaF family protein	--
WP_261897665.1	2	plus	type II secretion system F family protein	--
WP_261897666.1	2	plus	type II secretion system F family protein	--
WP_261897667.1	2	plus	hypothetical protein	--
WP_261897668.1	2	plus	TadE family protein	--

Type IV pili genes

Vp_protein_ID	Chr no.	Orientation	Annotation	*Symbol*
WP_261893862.1	1	minus	type IV pilus secretin PilQ	*pilQ*
WP_261893863.1	1	minus	hypothetical protein	*pilP*
WP_261893864.1	1	minus	type 4a pilus biogenesis protein PilO	*pilO*
WP_261893865.1	1	minus	PilN domain-containing protein	*pilN*
WP_261893867.1	1	minus	type IV pilus assembly protein PilM	*pilM*
WP_261893869.1	1	plus	PBP1A family penicillin-binding protein	*mrcA*
WP_261894724.1	1	plus	type IV pilus twitching motility protein PilT	*pilT*
WP_261894725.1	1	plus	PilT/PilU family type 4a pilus ATPase	*pilU*
WP_261892540.1	1	plus	type IV pilus biogenesis/stability protein PilW	*pilW*
WP_261893109.1	1	minus	FimV/HubP family polar landmark protein	*fimV*
WP_261893231.1	1	plus	type IV pilin protein	*pilE*
WP_261893587.1	1	minus	penicillin-binding protein 1B	*mrcB*
WP_315972737.1	1	plus	prepilin-type N-terminal cleavage/methylation domain-containing protein	*pilA*
WP_261893618.1	1	plus	ATPase, T2SS/T4P/T4SS family	*pilB*
WP_261893620.1	1	plus	type II secretion system F family protein	*pilC*
WP_261893621.1	1	plus	A24 family peptidase	*pilD*
WP_261894960.1	1	minus	PilZ domain-containing protein	--
WP_261892961.1	1	plus	PilZ domain-containing protein	--
WP_261893496.1	1	plus	PilZ domain-containing protein	--
WP_261893225.1	1	minus	prepilin-type N-terminal cleavage/methylation domain-containing protein	*PilV*
WP_261893226.1	1	minus	hypothetical protein	--
WP_261893227.1	1	minus	prepilin-type N-terminal cleavage/methylation domain-containing protein	*pilW*
WP_261893229.1	1	minus	GspH/FimT family pseudopilin	*FimT/FimU*

Lipopolysaccharides/LPS/Exopolysaccharides

WP_261893726.1	1	minus	LPS export ABC transporter permease LptG	*lptG*
WP_261893727.1	1	minus	LPS export ABC transporter permease LptF	*lptF*
WP_261893757.1	1	plus	arabinose-5-phosphate isomerase KdsD	*kdsD*
WP_261893758.1	1	plus	3-deoxy-manno-octulosonate-8-phosphatase KdsC	*kdsC*
WP_261893759.1	1	plus	LPS export ABC transporter periplasmic protein LptC	*lptC*
WP_261893761.1	1	plus	lipopolysaccharide transport periplasmic protein LptA	*lptA*
WP_261893763.1	1	plus	LPS export ABC transporter ATP-binding protein	*lptB*
WP_261894307.1	1	plus	glycosyltransferase family 9 protein	--
WP_261894310.1	1	minus	glycosyltransferase family 4 protein	--
WP_261894311.1	1	minus	glycosyltransferase family 2 protein	--
WP_261894312.1	1	minus	glycosyltransferase family 9 protein	--
WP_261896199.1	1	plus	3-deoxy-D-manno-octulosonic acid kinase	--

WP_261894313.1	1	plus	DUF3413 domain-containing protein	--
WP_261894314.1	1	plus	lipid IV(A) 3-deoxy-D-manno-octulosonic acid transferase	*waaA*
WP_261894316.1	1	plus	PIG-L family deacetylase	--
WP_261894317.1	1	minus	glycosyltransferase	--
WP_261894318.1	1	minus	O-antigen ligase family protein	--
WP_261894319.1	1	minus	lipopolysaccharide heptosyltransferase II	*waaF*
WP_261894320.1	1	minus	lauroyl-Kdo(2)-lipid IV(A) myristoyltransferase	*lpxM*
WP_261894322.1	1	minus	nucleotide sugar dehydrogenase	--
WP_261894324.1	1	minus	ADP-glyceromanno-heptose 6-epimerase	*rfaD*
WP_261894325.1	1	plus	ISAs1 family transposase	--
WP_261894328.1	1	plus	oligosaccharide flippase family protein	--
WP_261894329.1	1	plus	glycosyltransferase family 4 protein	--
WP_261894331.1	1	plus	hypothetical protein	--
WP_261894333.1	1	plus	O-antigen polymerase	--
WP_261894335.1	1	plus	hypothetical protein	--
WP_261894337.1	1	plus	glycosyltransferase	--
WP_261894338.1	1	plus	polysaccharide biosynthesis protein	--
WP_261894340.1	1	plus	SDR family oxidoreductase	--
WP_261894342.1	1	plus	UDP-N-acetylglucosamine 2-epimerase (non-hydrolyzing)	*wecB*
WP_261894344.1	1	plus	glycosyltransferase family 4 protein	--
WP_261894346.1	1	plus	WbuC family cupin fold metalloprotein	--
WP_261894348.1	1	minus	UDP-N-acetylglucosamine--undecaprenyl-phosphate N-acetylglucosaminephosphotransferase	*wecA*
WP_261894350.1	1	minus	ISAs1 family transposase	--
WP_261894351.1	1	minus	serine protease	--
WP_261894353.1	1	plus	polysaccharide export protein	--
WP_261894355.1	1	plus	low molecular weight phosphotyrosine protein phosphatase	--
WP_261894357.1	1	plus	polysaccharide biosynthesis tyrosine autokinase	--
WP_261894359.1	1	plus	oligosaccharide flippase family protein	--
WP_261894361.1	1	plus	glycosyltransferase family 2 protein	--
WP_261894363.1	1	plus	hypothetical protein	--
WP_261894365.1	1	plus	hypothetical protein	--
WP_261894367.1	1	plus	glycosyltransferase	--
WP_315972741.1	1	plus	acyltransferase	--
WP_261894369.1	1	plus	acyltransferase family protein	--
WP_261894370.1	1	plus	glycosyltransferase	--
WP_261894372.1	1	plus	3-deoxy-8-phosphooctulonate synthase	*kdsA*
WP_261894374.1	1	plus	3-deoxy-manno-octulosonate cytidylyltransferase	*kdsB*
WP_261894375.1	1	plus	KpsF/GutQ family sugar-phosphate isomerase	--
WP_261894377.1	1	plus	undecaprenyl-phosphate galactose phosphotransferase WbaP	*wbaP*
WP_261894379.1	1	plus	UTP--glucose-1-phosphate uridylyltransferase GalU	*galU*
WP_261894381.1	1	plus	hypothetical protein	--
WP_261894382.1	1	plus	polysaccharide biosynthesis/export family protein	--
WP_261894384.1	1	plus	ABC transporter permease	--
WP_261894386.1	1	plus	lipopolysaccharide biosynthesis protein	--
WP_261894387.1	1	plus	glycosyltransferase family 2 protein	--
WP_261894389.1	1	plus	glycosyltransferase family 2 protein	--
WP_261894391.1	1	plus	dTDP-glucose 4,6-dehydratase	*rfbB*
WP_261896200.1	1	plus	3-deoxy-8-phosphooctulonate synthase	*kdsA*
WP_261894401.1	1	plus	3-deoxy-manno-octulosonate cytidylyltransferase	*kdsB*
WP_261894402.1	1	plus	KpsF/GutQ family sugar-phosphate isomerase	--
WP_261894403.1	1	plus	hypothetical protein	--

WP_261894405.1	1	plus	HAD-IA family hydrolase	--
WP_261894406.1	1	plus	hypothetical protein	--
WP_261894408.1	1	plus	glycosyltransferase family 92 protein	--
WP_261894410.1	1	plus	ABC transporter ATP-binding protein	--
WP_261894422.1	1	plus	YjbF family lipoprotein	--
WP_261894424.1	1	plus	capsule biosynthesis GfcC family protein	--
WP_261894425.1	1	plus	YjbH domain-containing protein	--
WP_261894427.1	1	plus	dTDP-glucose 4,6-dehydratase	*rfbB*
WP_261894429.1	1	plus	glucose-1-phosphate thymidylyltransferase RfbA	*rfbA*
WP_261894431.1	1	plus	dTDP-4-dehydrorhamnose reductase	*rfbD*
WP_261894433.1	1	plus	dTDP-4-dehydrorhamnose 3,5-epimerase	*rfbC*
WP_261894640.1	1	minus	PglL family O-oligosaccharyltransferase	--
WP_261894643.1	1	minus	UTP--glucose-1-phosphate uridylyltransferase GalU	*galU*
WP_261894707.1	1	minus	peptidylprolyl isomerase SurA	*surA*
WP_261894709.1	1	minus	LPS assembly protein LptD	*lptD*
WP_261896206.1	1	minus	YqgE/AlgH family protein	--
WP_261895468.1	1	minus	glycosyltransferase family 9 protein	--
WP_261895892.1	1	plus	glycosyltransferase	--
WP_261892770.1	1	plus	adhesin biosynthesis transcription regulatory family protein	--
WP_261892839.1	1	plus	outer membrane beta-barrel protein	--
WP_261892982.1	1	minus	lipopolysaccharide assembly protein LapB	*lapB*
WP_261892983.1	1	minus	lipopolysaccharide assembly protein LapA domain-containing protein	--
WP_261893151.1	1	plus	apolipoprotein N-acyltransferase	*lnt*
WP_261893154.1	1	plus	LPS assembly lipoprotein LptE	*lptE*
WP_261896574.1	2	minus	exopolysaccharide biosynthesis protein	--
WP_261897107.1	2	plus	glycosyltransferase family 2 protein	--
WP_318757914.1	2	plus	GtrA family protein	--

Capsular protein

WP_261894305.1	1	plus	capsular polysaccharide synthesis protein	--

Cell surface anchoring polysaccharides

WP_261892669.1	1	minus	XrtA-associated ATPase	--
WP_261892671.1	1	minus	TIGR03016 family PEP-CTERM system-associated outer membrane protein	--
WP_261892672.1	1	minus	AAA family ATPase	--
WP_261892673.1	1	minus	Wzz/FepE/Etk N-terminal domain-containing protein	--
WP_261892674.1	1	minus	XrtA-associated exopolysaccharide export protein, Rfer_0658/Tmz1t_3282 family	--
WP_261892675.1	1	plus	TIGR03013 family PEP-CTERM/XrtA system glycosyltransferase	--
WP_261892676.1	1	plus	DUF3473 domain-containing protein	--
WP_261892677.1	1	plus	FemAB family PEP-CTERM system-associated protein	--
WP_261892678.1	1	plus	TIGR03087 family PEP-CTERM/XrtA system glycosyltransferase	--
WP_261892679.1	1	plus	exosortase A	*xrtA*
WP_261892680.1	1	plus	acyltransferase	--
WP_261892681.1	1	plus	amidotransferase 1, exosortase A system-associated	--
WP_261892682.1	1	plus	glycosyltransferase family 4 protein	--
WP_261892683.1	1	plus	glycosyltransferase family 4 protein	--
WP_261892684.1	1	minus	GNAT family N-acetyltransferase	--
WP_261892685.1	1	plus	LysR family transcriptional regulator	--
WP_261892686.1	1	minus	DMT family transporter	--

WP_261892687.1	1	plus	LysR substrate-binding domain-containing protein	--
WP_261892688.1	1	plus	hypothetical protein	--
WP_261892689.1	1	plus	SEC-C domain-containing protein	--
WP_261892690.1	1	minus	glycosyltransferase family A protein	--
WP_261892691.1	1	minus	putative O-glycosylation ligase, exosortase A system-associated	--
WP_261892692.1	1	minus	glycosyltransferase	--
WP_261892693.1	1	plus	GHMP kinase	--
WP_261892694.1	1	plus	nucleotidyltransferase family protein	--
WP_261892695.1	1	minus	polysaccharide pyruvyl transferase family protein	--
WP_261892696.1	1	minus	oligosaccharide flippase family protein	--
WP_261892697.1	1	minus	glycosyltransferase family 8 protein	--
WP_261892698.1	1	minus	glycosyltransferase	--
WP_261892699.1	1	minus	polysaccharide pyruvyl transferase family protein	--
WP_261892700.1	1	minus	hypothetical protein	--
WP_261892701.1	1	minus	ATP-grasp domain-containing protein	--
WP_261892702.1	1	minus	oligosaccharide flippase family protein	--
WP_261892703.1	1	minus	GNAT family N-acetyltransferase	--
WP_261892704.1	1	plus	PEP-CTERM system TPR-repeat protein PrsT	*prsT*
WP_261892705.1	1	plus	serine protease	--
WP_261892706.1	1	plus	VanZ family protein	--
WP_261892707.1	1	minus	ThiF family adenylyltransferase	--
WP_261892708.1	1	minus	hypothetical protein	--
WP_261892709.1	1	plus	PEP-CTERM sorting domain-containing protein	--
WP_261892710.1	1	plus	PEP-CTERM sorting domain-containing protein	--
WP_261892711.1	1	plus	PEP-CTERM system histidine kinase PrsK	*prsK*
WP_261892712.1	1	plus	PEP-CTERM-box response regulator transcription factor	*prsR*
WP_261892713.1	1	plus	MMPL family transporter	--
WP_261892714.1	1	plus	outer membrane lipoprotein-sorting protein	--
WP_261895394.1	1	minus	rhombosortase	*rrtA*
WP_261897045.1	2	minus	PEP-CTERM sorting domain-containing protein	--
WP_261897759.1	2	minus	PEP-CTERM sorting domain-containing protein	--

Expansin

WP_261897435.1	2	minus	expansin EXLX1 family cellulose-binding protein	--

Mannose-sensitive hemagglutinin (MSHA)

WP_261894654.1	1	plus	RNase E specificity factor CsrD	*mshH*
WP_261894655.1	1	plus	MSHA biogenesis protein MshI	*mshI*
WP_261894656.1	1	plus	type 4a pilus biogenesis protein PilO	*mshJ*
WP_261894657.1	1	plus	MSHA biogenesis protein MshK	*mshK*
WP_261894659.1	1	plus	pilus (MSHA type) biogenesis protein MshL	*mshL*
WP_261894660.1	1	plus	AAA family ATPase	*mshM*
WP_261894661.1	1	plus	MSHA biogenesis protein MshN	*mshN*
WP_261894662.1	1	plus	type II/IV secretion system protein	*mshE*
WP_261894664.1	1	plus	type II secretion system F family protein	*mshG*
WP_261894665.1	1	plus	MSHA biogenesis protein MshF	*mshF*
WP_261894666.1	1	plus	prepilin-type N-terminal cleavage/methylation domain-containing protein	*mshB*
WP_261896205.1	1	plus	prepilin-type N-terminal cleavage/methylation domain-containing protein	*mshA*
WP_261894667.1	1	plus	prepilin-type N-terminal cleavage/methylation domain-containing protein	*mshC*
WP_261894668.1	1	plus	type II secretion system protein	*mshD*

WP_261894670.1	1	plus	prepilin-type N-terminal cleavage/methylation domain-containing protein	*mshO*
WP_261894671.1	1	plus	MSHA biogenesis protein MshP	*mshP*
WP_261894673.1	1	plus	DUF6701 domain-containing protein	*mshQ*

(I) Iron acquisition and transport – TonB receptors, siderophore enzymes, heme utilization proteins, ferric citrate transport, enterobactins

Vp_protein_ID	Chr no.	Orientation	Annotation	Symbol
WP_261893123.1	1	minus	ferrochelatase	*hemH*
WP_261893248.1	1	minus	TonB-dependent siderophore receptor	--
WP_261893285.1	1	minus	TonB-dependent siderophore receptor	--
WP_261893286.1	1	minus	iron-siderophore ABC transporter substrate-binding protein	--
WP_261894007.1	1	minus	TonB-dependent receptor	
WP_261894101.1	1	minus	non-heme ferritin	*ftnA*
WP_261894279.1	1	plus	ABC transporter ATP-binding protein	--
WP_261894280.1	1	plus	iron-siderophore ABC transporter substrate-binding protein	--
WP_261894282.1	1	plus	Fe(3+)-hydroxamate ABC transporter permease FhuB	*fhuB*
WP_261894283.1	1	plus	TonB-dependent siderophore receptor	--
WP_261894478.1	1	minus	TonB-dependent receptor	
WP_261894611.1	1	plus	(2Fe-2S)-binding protein	--
WP_261894613.1	1	plus	bacterioferritin	*bfr*
WP_261895096.1	1	minus	Fe3+-citrate ABC transporter substrate-binding protein	--
WP_261895208.1	1	minus	ferric iron uptake transcriptional regulator FcrX	*fcrX*
WP_261895563.1	1	plus	FTR1 family protein	--
WP_261895565.1	1	plus	Fe-S-containing protein	--
WP_261895567.1	1	plus	ABC transporter permease	--
WP_261895610.1	1	minus	energy transducer TonB	*tonB*
WP_261895656.1	1	minus	ABC transporter substrate-binding protein	--
WP_261895658.1	1	plus	iron ABC transporter permease	--
WP_261895659.1	1	plus	ABC transporter ATP-binding protein	--
WP_261895884.1	1	plus	TonB-dependent siderophore receptor	--
WP_261895885.1	1	plus	ABC transporter substrate-binding protein	--
WP_261895887.1	1	plus	ABC transporter ATP-binding protein	--
WP_261895889.1	1	plus	iron ABC transporter permease	--
WP_261896111.1	1	plus	MotA/TolQ/ExbB proton channel family protein	*exbB*
WP_261896112.1	1	plus	MotA/TolQ/ExbB proton channel family protein	*exbB*
WP_261896113.1	1	plus	biopolymer transporter ExbD	*exbD*
WP_261896115.1	1	plus	energy transducer TonB	*tonB*
WP_261896117.1	1	plus	siderophore ferric iron reductase	--
WP_261896252.1	1	plus	hypothetical protein	--
WP_261896117.1	1	plus	siderophore ferric iron reductase	--
WP_261893236.1	1	plus	TonB-dependent receptor	--
WP_261893248.1	1	minus	TonB-dependent siderophore receptor	--
WP_261893285.1	1	minus	TonB-dependent siderophore receptor	--
WP_261893286.1	1	minus	iron-siderophore ABC transporter substrate-binding protein	--
WP_261896666.1	2	minus	TonB-dependent siderophore receptor	--
WP_261896815.1	2	plus	ATP-binding cassette domain-containing protein	--
WP_261896816.1	2	plus	iron-siderophore ABC transporter substrate-binding protein	--
WP_261896817.1	2	plus	Fe(3+)-hydroxamate ABC transporter permease FhuB	--
WP_261896818.1	2	plus	TonB-dependent receptor	--
WP_261897987.1	2	minus	TonB-dependent receptor	--
WP_261896976.1	2	plus	TonB-dependent siderophore receptor	--
WP_261897525.1	2	minus	TonB-dependent siderophore receptor	--
WP_261896373.1	2	plus	TonB-dependent hemoglobin/transferrin/lactoferrin family receptor	--

WP_261896432.1	2	plus	TonB-dependent receptor plug domain-containing protein	--
WP_261892600.1	1	plus	ABC transporter substrate-binding protein	--
WP_261892602.1	1	plus	iron ABC transporter permease	--
WP_261892604.1	1	plus	ABC transporter ATP-binding protein	--
				--
WP_261897037.1	2	minus	ABC transporter substrate-binding protein	--
WP_261897038.1	2	minus	ABC transporter ATP-binding protein	--
WP_261897039.1	2	minus	iron ABC transporter permease	--
WP_261897167.1	2	plus	ABC transporter substrate-binding protein	--
WP_261897168.1	2	plus	iron ABC transporter permease	--
WP_261897169.1	2	plus	ABC transporter ATP-binding protein	--
WP_261897222.1	2	plus	Fe(3+) dicitrate ABC transporter substrate-binding protein	--
WP_261897223.1	2	plus	Fe(3+) dicitrate ABC transporter substrate-binding protein	--
WP_261897224.1	2	plus	iron chelate uptake ABC transporter family permease subunit	--
WP_261897225.1	2	plus	Fe(3+) dicitrate ABC transporter permease subunit FecD	*fecD*
WP_261897226.1	2	plus	Fe(3+) dicitrate ABC transporter ATP-binding protein FecE	*fecE*
WP_261897406.1	2	minus	heme ABC transporter ATP-binding protein	--
WP_261897952.1	2	minus	iron ABC transporter permease	--
WP_261897407.1	2	minus	ABC transporter substrate-binding protein	--
WP_261897408.1	2	plus	heme anaerobic degradation radical SAM methyltransferase ChuW/HutW	*hutW*
WP_261897409.1	2	plus	heme utilization cystosolic carrier protein HutX	*hutX*
WP_261897410.1	2	plus	heme utilization protein HutZ	*hutZ*
WP_261897525.1	2	minus	TonB-dependent siderophore receptor	--
WP_261897714.1	2	minus	siderophore-interacting protein	--
WP_261897715.1	2	plus	extracellular solute-binding protein	--
WP_261896373.1	2	plus	TonB-dependent hemoglobin/transferrin/lactoferrin family receptor	--
WP_261896432.1	2	plus	TonB-dependent receptor plug domain-containing protein	--
WP_261896506.1	2	minus	iron chelate ABC transporter ATP-binding protein VctC	*vctC*
WP_261896507.1	2	minus	iron chelate uptake ABC transporter permease subunit VctG	*vctG*
WP_261896508.1	2	minus	iron chelate uptake ABC transporter permease subunit VctD	*vctD*
WP_261896509.1	2	minus	siderophore ABC transporter substrate-binding protein	--
WP_261895393.1	1	minus	enterochelin esterase	*fes*
WP_261897771.1	2	minus	alpha/beta hydrolase-fold protein	*fes*
WP_261897772.1	2	minus	2,3-dihydro-2,3-dihydroxybenzoate dehydrogenase	*entA*
WP_261897773.1	2	plus	isochorismate synthase	*entC*
WP_261897774.1	2	plus	(2,3-dihydroxybenzoyl)adenylate synthase	*entE*
WP_261897775.1	2	plus	isochorismatase family protein	*entB1*
WP_261897776.1	2	plus	4'-phosphopantetheinyl transferase superfamily protein	*entD*
WP_261897777.1	2	plus	MbtH family protein	*mtbH*
WP_261897778.1	2	plus	amino acid adenylation domain-containing protein	*entF*
WP_261897779.1	2	plus	enterobactin transporter EntS	*entS*

(m) Embden-Meyerhof-Parnas pathway

Vp_protein_ID	Chr no.	Orientation	Annotation	Symbol
WP_261893912.1	1	plus	triose-phosphate isomerase	*tpiA*
WP_261893927.1	1	minus	6-phosphofructokinase	*pfkA*
WP_261894549.1	1	minus	2,3-bisphosphoglycerate-independent phosphoglycerate mutase	*gpmM*
WP_261897211.1	2	minus	glucokinase	*glk*
WP_261894622.1	1	plus	glucose-6-phosphate isomerase	*pgi*

WP_261894736.1	1	plus	phosphoglycerate kinase	--
WP_261894737.1	1	plus	class II fructose-bisphosphate aldolase	*fbaA*
WP_261894746.1	1	minus	pyruvate kinase PykF	*pykF*
WP_261895369.1	1	minus	pyruvate kinase	*pyk*
WP_261892826.1	1	minus	ArsJ-associated glyceraldehyde-3-phosphate dehydrogenase	--
WP_261893043.1	1	plus	glyceraldehyde-3-phosphate dehydrogenase	*gapA*
WP_261893662.1	1	minus	phosphopyruvate hydratase	*eno*

(n) Entner-Doudoroff and pentose phosphate pathways

Vp_protein_ID	Chr no.	Orientation	Annotation	Symbol
WP_261894732.1	1	plus	transketolase	*tkt*
WP_261893699.1	1	plus	ribose-5-phosphate isomerase RpiA	*rpiA*
WP_261893856.1	1	minus	ribulose-phosphate 3-epimerase	*rpe*
WP_261896964.1	2	plus	bifunctional 4-hydroxy-2-oxoglutarate aldolase/2-dehydro-3-deoxy-phosphogluconate aldolase	--
WP_261897211.1	2	minus	glucokinase	*glk*
WP_261897345.1	2	minus	bifunctional 4-hydroxy-2-oxoglutarate aldolase/2-dehydro-3-deoxy-phosphogluconate aldolase	--
WP_261897419.1	2	minus	decarboxylating NADP(+)-dependent phosphogluconate dehydrogenase	*gnd*
WP_261897420.1	2	minus	6-phosphogluconolactonase	*pgl*
WP_261897953.1	2	minus	glucose-6-phosphate dehydrogenase	*zwf*
WP_261897502.1	2	minus	glucokinase	*glk*
WP_261897699.1	2	minus	transketolase	*tkt*
WP_261897700.1	2	minus	transaldolase	*tal*

(o) TCA cycle

Vp_protein_ID	Chr no.	Orientation	Annotation	Symbol
WP_261894693.1	1	plus	malate dehydrogenase	*mdh*
WP_261895220.1	1	minus	citrate synthase	--
WP_261895222.1	1	plus	succinate dehydrogenase cytochrome b556 subunit	*sdhC*
WP_261895223.1	1	plus	succinate dehydrogenase, hydrophobic membrane anchor protein	*sdhD*
WP_261895224.1	1	plus	succinate dehydrogenase flavoprotein subunit	*sdhA*
WP_261895226.1	1	plus	succinate dehydrogenase iron-sulfur subunit	--
WP_261896226.1	1	plus	2-oxoglutarate dehydrogenase E1 component	*sucA*
WP_261895227.1	1	plus	2-oxoglutarate dehydrogenase complex dihydrolipoyllysine-residue succinyltransferase	*odhB*
WP_261895229.1	1	plus	ADP-forming succinate--CoA ligase subunit beta	*sucC*
WP_261895231.1	1	plus	succinate--CoA ligase subunit alpha	*sucD*
WP_261895752.1	1	plus	NADP-dependent isocitrate dehydrogenase	--
WP_261895503.1	1	plus	aconitase family protein	--
WP_261896067.1	1	plus	fumarate hydratase	--
WP_261893608.1	1	minus	dihydrolipoyl dehydrogenase	*lpdA*
WP_261893610.1	1	minus	pyruvate dehydrogenase complex dihydrolipoyllysine-residue acetyltransferase	*aceF*
WP_261893611.1	1	minus	pyruvate dehydrogenase (acetyl-transferring), homodimeric type	*aceE*
WP_261893613.1	1	minus	pyruvate dehydrogenase complex transcriptional repressor PdhR	*pdhR*
WP_261896067.1	1	plus	fumarate hydratase	--
WP_261896098.1	1	plus	fumarylacetoacetate hydrolase family protein	--

(p) Glyoxylate shunt pathway

Vp_protein_ID	Chr no.	Orientation	Annotation	Symbol
WP_261893461.1	1	minus	isocitrate lyase	*aceA*
WP_261893462.1	1	minus	malate synthase A	*aceB*

(q) Monosaccharide, sugar alcohol, dicarboxylic acids operons

Vp_protein_ID	Chr no.	Orientation	Annotation	Symbol
**Arabinose**				
WP_261894079.1	1	minus	arabinose operon transcriptional regulator AraC	*araC*
WP_261894081.1	1	minus	hypothetical protein	--
WP_261894083.1	1	minus	L-arabinose isomerase	*araA*
WP_315972738.1	1	minus	L-ribulose-5-phosphate 4-epimerase	*araD*
WP_261894084.1	1	minus	ribulokinase	*araB*
WP_261894086.1	1	plus	arabinose ABC transporter substrate-binding protein	*araF*
WP_261894088.1	1	plus	L-arabinose ABC transporter ATP-binding protein AraG	*araG*
WP_315972739.1	1	plus	L-arabinose ABC transporter permease AraH	*araH*
**Rhamnose**				
WP_261895836.1	1	plus	rhamnulokinase	*rhaB*
WP_261895838.1	1	plus	L-rhamnose isomerase	*rhaA*
WP_261895840.1	1	plus	L-rhamnose/proton symporter RhaT	*rhaT*
WP_261895842.1	1	plus	L-rhamnose mutarotase	*rhaM*
WP_261895844.1	1	plus	rhamnulose-1-phosphate aldolase	*rhaD*
WP_261895846.1	1	plus	HTH-type transcriptional activator	*rhaS*
**Xylose**				
WP_261893317.1	1	minus	xylose isomerase	*xylA*
WP_261893319.1	1	plus	sugar porter family MFS transporter	*xylE*
WP_261893320.1	1	minus	DNA-binding transcriptional regulator	*xylR*
WP_261896301.1	1	plus	xylulokinase	*xylB*
**Fructose**				
**cluster 1**				
WP_261896407.1	2	minus	catabolite repressor/activator	*fruR*
WP_261896408.1	2	plus	fused PTS fructose transporter subunit IIA/HPr protein	*fruB*
WP_261896409.1	2	plus	1-phosphofructokinase	*fruK*
WP_261896410.1	2	plus	PTS fructose transporter subunit IIBC	*fruA*
**Cluster 2**				
WP_261896789.1	2	minus	D-allulose 6-phosphate 3-epimerase (converts allose to fructose)	*alsE*
WP_261896790.1	2	minus	hypothetical protein	*frwC*
WP_261896791.1	2	minus	PTS fructose transporter subunit IIC	*frwC*
WP_261896792.1	2	minus	PTS fructose transporter subunit IIB	*frwB*
WP_261896793.1	2	minus	fructose PTS transporter subunit IIA	*frwA*
WP_261896794.1	2	minus	PRD domain-containing protein	*BglG regulator*
WP_261893927.1	1	minus	6-phosphofructokinase	*pfkA*
**myo-inositol**				
WP_261896723.1	2	plus	CoA-acylating methylmalonate-semialdehyde dehydrogenase	*iolA*
WP_261896724.1	2	plus	5-deoxy-glucuronate isomerase	*iolB*
WP_261896725.1	2	minus	NAD(P)-dependent alcohol dehydrogenase	--

WP_261896726.1	2	minus	MurR/RpiR family transcriptional regulator	*iolR*
WP_261896727.1	2	minus	5-dehydro-2-deoxygluconokinase	*iolC*
WP_261896728.1	2	plus	3D-(3,5/4)-trihydroxycyclohexane-1,2-dione acylhydrolase (decyclizing)	*iolD*
WP_261896729.1	2	plus	sugar phosphate isomerase/epimerase	*iolH*
WP_261896001.1	1	plus	Gfo/Idh/MocA family oxidoreductase (Inositol 2 dehydrogenase like)	--
**Mannitol**				
WP_261897190.1	2	minus	MltR family transcriptional regulator	*mltR*
WP_261897191.1	2	minus	mannitol-1-phosphate 5-dehydrogenase	*mltD*
WP_261897192.1	2	minus	PTS mannitol transporter subunit IICBA	*mltA*
**Ribose**				
WP_261897281.1	2	plus	D-ribose pyranase	*rbsD*
WP_261897282.1	2	plus	ribose ABC transporter ATP-binding protein RbsA	*rbsA*
WP_261897283.1	2	plus	ribose ABC transporter permease	*rbsC*
WP_261897284.1	2	plus	ribose ABC transporter substrate-binding protein RbsB	*rbsB*
WP_261897285.1	2	plus	ribokinase	*rbsK*
WP_261897286.1	2	plus	substrate-binding domain-containing protein	*rbsR*
**Dicarboxylic acids**				
**Malonate**				
WP_261895518.1	1	minus	LysR family transcriptional regulator	*mdcR*
WP_261895520.1	1	minus	malonate decarboxylase subunit epsilon	*mdcH*
WP_261895521.1	1	minus	malonate decarboxylase holo-ACP synthase	*mdcG*
WP_261895522.1	1	minus	AEC family transporter	*yfdV*
WP_261895523.1	1	minus	biotin-independent malonate decarboxylase subunit gamma	*mdcE*
WP_261895525.1	1	minus	biotin-independent malonate decarboxylase subunit beta	*mdcD*
WP_261895527.1	1	minus	triphosphoribosyl-dephospho-CoA synthase	*mdcB*
WP_261895528.1	1	minus	malonate decarboxylase subunit alpha	*mdcA*
**Gluconate**				
WP_261896327.1	1	minus	gluconokinase	*gntK*
WP_261894096.1	1	plus	gluconate transporter	*gntU*
WP_261894097.1	1	minus	gluconate operon transcriptional repressor GntR	*gntR*
**Galactarate/glucarate**				
WP_261897413.1	2	plus	galactarate dehydratase	*garD*
WP_261897414.1	2	minus	sugar diacid recognition domain-containing protein	*cdaR*
WP_261896847.1	2	minus	glycerate kinase	*glxK*
WP_261896848.1	2	minus	2-hydroxy-3-oxopropionate reductase	*garR*
WP_261896849.1	2	minus	2-dehydro-3-deoxyglucarate aldolase	*garL*
WP_261896448.1	2	plus	glucarate dehydratase family protein	*gudD*

(r) CAZymes

Vp_protein_ID	Chr no.	Orientation	Annotation	CAZy family
WP_318757729.1	2	plus	family 43 glycosylhydrolase	CBM13,GH43
WP_261897623.1	2	plus	RICIN domain-containing protein	CBM13,PL1
WP_261892324.1	1	minus	RICIN domain-containing protein	CBM13,PL9
WP_261897106.1	2	plus	glycogen debranching protein GlgX	CBM48,GH13
WP_261897133.1	2	minus	1,4-alpha-glucan branching protein GlgB	CBM48,GH13

WP_261896207.1	1	plus	peptidoglycan DD-metalloendopeptidase family protein	CBM50
WP_261897128.1	2	minus	LysM domain-containing protein	CBM50
WP_261894573.1	1	plus	N-acetylmuramoyl-L-alanine amidase	CBM50,CBM50,CBM50
WP_261896599.1	2	minus	arabinogalactan endo-1,4-beta-galactosidase	CBM61,GH53
WP_261897435.1	2	minus	expansin EXLX1 family cellulose-binding protein	CBM63
WP_261894896.1	1	plus	UDP-3-O-acyl-N-acetylglucosamine deacetylase	CE11
WP_261896606.1	2	plus	GDSL-type esterase/lipase family protein	CE2
WP_261897923.1	2	minus	allantoinase PuuE	CE4
WP_261896719.1	2	plus	pectinesterase family protein	CE8
WP_261892634.1	1	minus	putative acyl-CoA thioester hydrolase	CE8
WP_261897837.1	2	minus	pectinesterase family protein	CE8
WP_261897341.1	2	plus	pectinesterase family protein	CE8,CE12
WP_261893112.1	1	plus	N-acetylglucosamine-6-phosphate deacetylase	CE9
WP_261895665.1	1	minus	6-phospho-beta-glucosidase	GH1
WP_261896624.1	2	minus	family 1 glycosylhydrolase	GH1
WP_261895308.1	1	plus	glycoside hydrolase family 1 protein	GH1
WP_261897629.1	2	plus	6-phospho-beta-glucosidase	GH1
WP_261894459.1	1	plus	6-phospho-beta-glucosidase	GH1
WP_261892408.1	1	minus	6-phospho-beta-glucosidase	GH1
WP_261897028.1	2	minus	glycoside hydrolase family 1 protein	GH1
WP_261896689.1	2	plus	glycoside hydrolase family 1 protein	GH1
WP_261892385.1	1	minus	glycoside hydrolase family 1 protein	GH1
WP_261894986.1	1	plus	murein transglycosylase A	GH102
WP_261893023.1	1	minus	lytic murein transglycosylase	GH103
WP_261897348.1	2	minus	glycoside hydrolase family 88 protein	GH105
WP_261896609.1	2	plus	glycosyl hydrolase	GH106
WP_261897034.1	2	plus	glycosyl hydrolase	GH106
WP_261892907.1	1	plus	endo alpha-1,4 polygalactosaminidase	GH114
WP_261897243.1	2	minus	glycoside hydrolase family protein	GH128
WP_261895305.1	1	minus	glycosyl hydrolase	GH128,CBM6
WP_261897003.1	2	minus	alpha-amylase family glycosyl hydrolase	GH13
WP_261892831.1	1	minus	family 16 glycosylhydrolase	GH16,CBM6
WP_261892280.1	1	minus	glycosyl hydrolase family 18 protein	GH18
WP_261895598.1	1	plus	beta-galactosidase	GH2
WP_318757666.1	1	minus	transglycosylase SLT domain-containing protein	GH23
WP_261893215.1	1	plus	membrane-bound lytic murein transglycosylase MltF	GH23
WP_261893494.1	1	minus	transglycosylase SLT domain-containing protein	GH23
WP_261894711.1	1	minus	membrane-bound lytic murein transglycosylase MltC	GH23
WP_261895075.1	1	minus	LysM peptidoglycan-binding domain-containing protein	GH23,CBM50,CBM50, CBM50
WP_261892748.1	1	minus	lysozyme	GH24
WP_261894752.1	1	minus	glycoside hydrolase family 28 protein	GH28
WP_261893006.1	1	minus	glycoside hydrolase family 3 C-terminal domain-containing protein	GH3
WP_261896955.1	2	minus	glycoside hydrolase family 3 N-terminal domain-containing protein	GH3
WP_261896952.1	2	plus	glycoside hydrolase family 3 C-terminal domain-containing protein	GH3
WP_261895590.1	1	minus	glycoside hydrolase family 3 C-terminal domain-containing protein	GH3
WP_261897442.1	2	minus	glycoside hydrolase family 3 N-terminal domain-containing protein	GH3
WP_261893501.1	1	minus	beta-N-acetylhexosaminidase	GH3
WP_261895593.1	1	minus	glycoside hydrolase family 3 C-terminal domain-containing protein	GH3
WP_261896047.1	1	plus	hypothetical protein	GH30
WP_261895694.1	1	plus	glycoside hydrolase family 32 protein	GH32
WP_261896602.1	2	minus	beta-galactosidase	GH35
WP_261895600.1	1	plus	alpha-galactosidase	GH36
WP_261896042.1	1	plus	beta-galactosidase	GH42
WP_261897538.1	2	minus	beta-galactosidase	GH42

WP_261893008.1	1	minus	glycoside hydrolase family 43 protein	GH43
WP_261897006.1	2	plus	arabinan endo-1,5-alpha-L-arabinosidase	GH43
WP_261897722.1	2	plus	family 43 glycosylhydrolase	GH43,CBM13
WP_261897746.1	2	minus	hypothetical protein	GH5
WP_261897056.1	2	minus	cellulase family glycosylhydrolase	GH5,CBM2
WP_261896041.1	1	plus	glycosyl hydrolase 53 family protein	GH53
WP_261896044.1	1	plus	arabinogalactan endo-1,4-beta-galactosidase	GH53
WP_261895128.1	1	plus	flagellar assembly peptidoglycan hydrolase FlgJ	GH73
WP_261897134.1	2	minus	4-alpha-glucanotransferase	GH77
WP_261896435.1	2	minus	glycoside hydrolase family 78 protein	GH78
WP_261896144.1	1	plus	glucosaminidase domain-containing protein	GHnc
WP_261896208.1	1	plus	hypothetical protein	GHnc
WP_261893706.1	1	minus	MJ1255/VC2487 family glycosyltransferase	GT1
WP_261894886.1	1	plus	cell division protein FtsW	GT119
WP_261895068.1	1	plus	lipid-A-disaccharide synthase	GT19
WP_261892698.1	1	minus	glycosyltransferase	GT2
WP_261895892.1	1	plus	glycosyltransferase	GT2
WP_261894389.1	1	plus	glycosyltransferase family 2 protein	GT2
WP_261894361.1	1	plus	glycosyltransferase family 2 protein	GT2
WP_261893716.1	1	plus	glycosyltransferase family 2 protein	GT2
WP_261894311.1	1	minus	glycosyltransferase family 2 protein	GT2
WP_261897107.1	2	plus	glycosyltransferase family 2 protein	GT2
WP_261894387.1	1	plus	glycosyltransferase family 2 protein	GT2
WP_261894367.1	1	plus	glycosyltransferase	GT2
WP_261894888.1	1	plus	undecaprenyldiphospho-muramoylpentapeptide beta-N-acetylglucosaminyltransferase	GT28
WP_261894314.1	1	plus	lipid IV(A) 3-deoxy-D-manno-octulosonic acid transferase	GT30
WP_261894305.1	1	plus	capsular polysaccharide synthesis protein	GT32
WP_261897135.1	2	minus	glycogen/starch/alpha-glucan phosphorylase	GT35
WP_261892683.1	1	plus	glycosyltransferase family 4 protein	GT4
WP_261894337.1	1	plus	glycosyltransferase	GT4
WP_261894329.1	1	plus	glycosyltransferase family 4 protein	GT4
WP_261892682.1	1	plus	glycosyltransferase family 4 protein	GT4
WP_261894370.1	1	plus	glycosyltransferase	GT4
WP_261894344.1	1	plus	glycosyltransferase family 4 protein	GT4
WP_261894310.1	1	minus	glycosyltransferase family 4 protein	GT4
WP_261894317.1	1	minus	glycosyltransferase	GT4
WP_261892692.1	1	minus	glycosyltransferase	GT4
WP_261896240.1	1	plus	glycogen synthase GlgA	GT5
WP_261893587.1	1	minus	penicillin-binding protein 1B	GT51
WP_225251730.1	1	minus	monofunctional biosynthetic peptidoglycan transglycosylase	GT51
WP_261893869.1	1	plus	PBP1A family penicillin-binding protein	GT51
WP_261892697.1	1	minus	glycosyltransferase family 8 protein	GT8
WP_261894312.1	1	minus	glycosyltransferase family 9 protein	GT9
WP_261894319.1	1	minus	lipopolysaccharide heptosyltransferase II	GT9
WP_261892678.1	1	plus	TIGR03087 family PEP-CTERM/XrtA system glycosyltransferase	GTnc
WP_261894307.1	1	plus	glycosyltransferase family 9 protein	GTnc
WP_261895468.1	1	minus	glycosyltransferase family 9 protein	GTnc
WP_261896796.1	2	plus	hypothetical protein	PL1
WP_261896720.1	2	plus	polysaccharide lyase	PL1
WP_261894284.1	1	plus	hypothetical protein	PL1
WP_261897815.1	2	plus	polysaccharide lyase	PL1
WP_261895499.1	1	minus	RICIN domain-containing protein	PL1,CBM13
WP_261897333.1	2	minus	pectate lyase	PL2
WP_261897330.1	2	minus	oligogalacturonate lyase family protein	PL22
WP_261893328.1	1	plus	pectate lyase	PL3
WP_261892593.1	1	plus	pectate lyase	PL3
WP_261896782.1	2	minus	rhamnogalacturonan lyase B N-terminal domain-containing protein	PL4

WP_261897729.1	2	minus	polysaccharide lyase family protein	PL4
WP_261897339.1	2	plus	exopolygalacturonate lyase	PL9
WP_261897582.1	2	plus	right-handed parallel beta-helix repeat-containing protein	PL9
WP_261897547.1	2	minus	DUF4990 domain-containing protein	PL9
WP_261895677.1	1	plus	fibronectin type III domain-containing protein	PL9
